# How Population Structure and Nest Membership Shape Pathogen Patterns in Bumble Bees

**DOI:** 10.1111/mec.70146

**Published:** 2025-10-14

**Authors:** Jana Dobelmann, Lena Wilfert

**Affiliations:** ^1^ Institute of Evolutionary Ecology and Conservation Genomics University of Ulm Ulm Germany

**Keywords:** bumble bee, density, genetic diversity, pathogen, population structure

## Abstract

Host density, genetic diversity and social groups are key factors influencing pathogen transmission in wildlife populations, but their interactions remain poorly understood in insects. Islands can provide natural laboratories with distinct populations that vary in density and genetic diversity, whereby dense, genetically homogeneous populations are expected to facilitate pathogen transmission. We used bumble bees to test these predictions, assessing the population structure of the two common species *Bombus pascuorum* and *B. terrestris* across island and mainland sites in the British Isles and France and testing bees for five micro‐parasitic and four viral pathogens. *B. pascuorum* formed distinct genetic clusters on islands, with varying levels of heterozygosity and only the Isle of Arran clustered with mainland populations. *B. terrestris* populations were less structured, but populations on the Isle of Man and the Scilly Isles were genetically separated from other island and mainland populations while showing low heterozygosity. Colony density was similar between species and not linked to genetic diversity but had a positive effect on the prevalence of some pathogens. Contrary to expectations, there was no protective effect of high genetic diversity, suggesting that generalist bumble bee pathogens could be more affected by host species diversity and density. Yet, within *B. terrestris* populations, we found that nestmates showed more similar pathogen profiles than unrelated individuals, suggesting that genetic similarity and high contact rates within nests affect pathogen prevalence in wild bees.

## 1 Introduction

Understanding how population structure is affecting pathogen transmission is key for managing and predicting outbreaks across species. Population density, genetic diversity and interactions within groups can all shape the dynamics of pathogen spread. In dense populations, high contact rates can facilitate transmission, as exemplified by increased SARS‐CoV‐2 transmission rates in densely populated areas during the COVID‐19 pandemic (Sy et al. 2021) or the increased pathogen prevalence in birds at supplemental feeding sites (Lawson et al. 2018; Wilcoxen et al. 2015). While larger host populations increase transmission, they generally show higher phenotypic diversity in disease resistance (i.e., functional diversity) that can buffer against disease spread (King and Lively 2012; Lively 2010). Support for this link between genetic diversity and disease resistance comes from various research areas (Gibson 2022). For example, rice monocultures show lower yields and reduced resistance to fungal pathogens than seed mixtures (Zhu et al. 2000). But this monoculture effect is also found in seminatural populations of the crustacean *Daphnia*, where experiments found that a microsporidian parasite spread more easily in low‐ than in high‐diversity populations and maintained a higher prevalence (Altermatt and Ebert 2008). While the link between genetic diversity and reduced parasitism holds true in experimental host populations, this link appears inconsistent in natural host populations (Gibson and Nguyen 2021).

In addition to genetic diversity at the population level, pathogen transmission in group‐living animals can also be affected by the group's genetic diversity. Social insects that cohabit in high‐density colonies with closely related individuals would be expected to create conditions that facilitate pathogen transmission (Anderson et al. 1986; Schmid‐Hempel 1998; Shykoff and Schmid‐Hempel 1991). And indeed, within‐colony relatedness has been shown to increase the risk of disease and colony death in honey bees (Tarpy et al. 2013), bumble bees (Baer and Schmid‐Hempel 1999) and leaf‐cutting ants (Hughes and Boomsma 2004). Yet, social insects have evolved an array of behaviours that limit disease spread, including hygienic measures and organisational structures (Cremer et al. 2007). Another aspect of social groups is increased contact rates within groups, so that social structure and the size of subgroups within a population can affect transmission (Nunn et al. 2015), particularly in highly transmissible pathogens (Sah et al. 2018). So far, few studies have linked population‐level genetic diversity and social group membership, or in the case of social insects, nest membership, to pathogen dynamics.

Bumble bees can provide a useful model to study this link in wild populations. Many bumble bee species are distributed across large geographical regions, with populations varying in density and genetic diversity. Population structuring and reduced genetic diversity are characteristic of declining bumble bee species with small population sizes (Cameron et al. 2011; Ellis et al. 2006; Goulson et al. 2008; Maebe et al. 2019) that have been linked to agricultural intensification and human land use (Glück et al. 2022; Hemberger et al. 2021; Jha 2015). But even common species can, in addition to recent anthropogenic effects, show structuring that is affected by their historical geographical distribution. Bottlenecks during glaciation and, in the northern hemisphere, recolonisation from southern refugia have shaped their genetic diversity (Kelemen and Rehan 2021; Ranjbaran et al. 2024). Additionally, natural barriers, such as mountains and oceans, limit gene flow (Schleimer and Frantz 2025). The Alps, for instance, separate bumble bees into genetic clusters (Pirounakis et al. 1998; Widmer and Schmid‐Hempel 1999) and island populations commonly exhibit lower heterozygosity and allelic richness compared to mainland populations (Jha 2015; Lozier et al. 2011). The distance to other islands or the mainland affects population connectivity (Darvill et al. 2010) and, with lower rates of gene flow, may also affect how disease resistance is distributed, potentially making isolated host populations more vulnerable to infection (Höckerstedt et al. 2022). Although common generalist bumble bee species on the European continent show little (Estoup et al. 1996; Widmer and Schmid‐Hempel 1999) or very weak (Glück et al. 2022) population structure and high connectivity, these populations could help reveal factors that shape population structure and allow linking these to pathogen prevalence. Multiple studies have found that genetic diversity in bumble bee populations negatively correlates with infections with the trypanosome *Crithidia bombi* (Parsche and Lattorff 2018; Shykoff and Schmid‐Hempel 1991; Whitehorn et al. 2011). Dense populations, on the other hand, show increased *C. bombi* infections (Parsche and Lattorff 2018), and experimentally increased bumble bee densities lead to increased prevalence of slow bee paralysis virus (SBPV) (Bailes et al. 2020). Additionally, increased pathogen prevalence in urban bumble bees has been linked to their higher density when compared to rural areas (Goulson et al. 2012; Theodorou et al. 2016), suggesting that population density, in addition to genetic diversity, plays a key role in bumble bee pathogen transmission.

Here, we use microsatellite markers to study the population structure and nest membership of two common bumble bee species, *Bombus pascuorum* and *B. terrestris* , across multiple islands and mainland sites on the British Isles and in France, that we then link to pathogen prevalence. Firstly, we examine how islands and geographic location affect population structure and distribution of genetic diversity in bees. Secondly, we estimate colony densities on islands to test whether high‐density populations with low genetic diversity have increased pathogen prevalence. Thirdly, we examine pathogen profiles within populations. We hypothesise that genetic similarity and close contact within the nest environment facilitate pathogen transmission and test whether bees are more similar in their pathogen profile when they originate from the same nest. Understanding factors that shape population structure and how this structure links to pathogen dynamics is important for disease management and pollinator conservation.

## 2 Materials and Methods

### 2.1 Sample Collection and DNA Extraction

**FIGURE 1:**
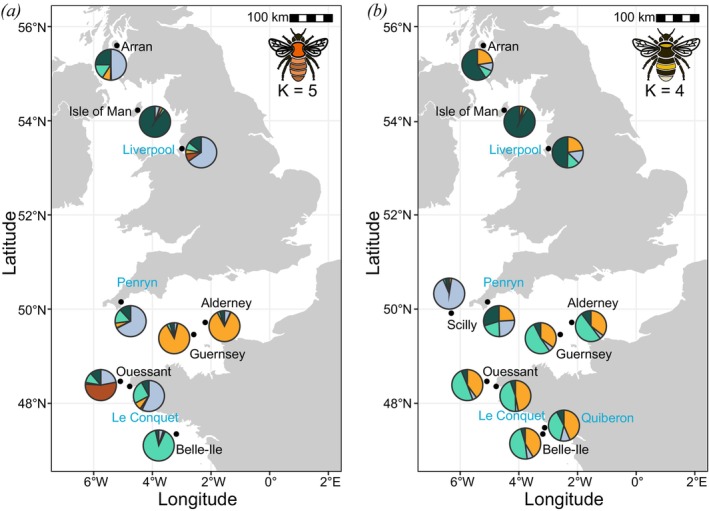
Population structure of (a) *Bombus pascuorum* and (b) *B. terrestris* on the British Isles and in France, shown as mean cluster membership. Pie charts show the average membership across ten subsampled data sets with 15 individuals as estimated by STRUCTURE. Results are shown for five genetic clusters (*K* = 5) in *B. pascuorum* and four (*K* = 4) in *B. terrestris* . Islands are shown in black font and mainland sites in blue. Detailed sampling locations on the six islands sampled in 2022 are shown in Figure S1.

To examine the population structure of foraging bumble bees, 216 *B* and 573 *B. terrestris* workers were collected from flowers from mid June to late August in 2021 in seminatural and suburban habitats across seven islands in the Irish and Celtic Sea, the English Channel and off the coast of Brittany in France, as well as from four coastal sites in mainland England and France (Figure 1, Figure S1, Table S1). *B. pascuorum* is absent on the Scilly Isles, and no or very few (4) individuals were found in Quiberon and on Belle‐Ile, respectively. From late June to early August 2022, islands with both species were sampled again to examine how population structure affects pathogen prevalence. A total of 988 *B. pascuorum* and 1163 *B. terrestris* were obtained that year. For this, five sampling sites were chosen on large islands (Arran, Isle of Man, Guernsey, Belle‐Ile) and on smaller islands (< 15 km^2^, Alderney and Ouessant) samples were collected across the whole island (Figure S1, Table S1). St Peter Port and Saumarez Park on Guernsey were the closest sampling sites (3.4 km apart), and the closest islands were Alderney and Guernsey (30 km apart). Bees were refrigerated upon collection and frozen within 24 h in a dry shipper before being transported to the lab, where they were stored at −80°C. Alderney was sampled twice in 2022, once in late June and again in early August. The samples collected in late June were defrosted during transit and had to be excluded from pathogen analyses but were used for population genetics.

DNA was extracted from a single hind leg crushed in liquid nitrogen using a pestle and 200 μL 5% Chelex solution and 2 μL proteinase K (Roche). Incubation was at 56°C for 1 h with shaking and inactivation at 96°C for 15 min. Extracts were centrifuged for 1 min at 21,300 ***g***, and 150 μL supernatant was removed and stored at −20°C. *B. terrestris* was distinguished from the co‐occurring and morphologically similar *B. lucorum* complex (Wolf et al. 2010) by polymerase chain reactions (PCR) with species‐specific length polymorphism (details in Appendix S1), resulting in 28 *B. lucorum* in 2021 and 270 in 2022 being excluded from the analysis. Most *B. lucorum* were found on Arran (211 in total) and the Isle of Man (48). In 2022, only one *B. terrestris* was caught at the northernmost site of Lochranza on the Isle of Arran and removed from the analysis.

### 2.2 Genotyping

The development of microsatellite markers has facilitated population structure analysis in bumble bees, as they can be used to group workers into sibships and these sibships can be used to estimate the number of colonies. Bumble bees were genotyped at 12 loci (Reber Funk et al. 2006; Wood et al. 2015) (details in Appendix S2, Table S2) and fragments were run on a Thermo Fisher Scientific Genetic Analyser 3130 Series using 9.7 μL Hi‐Di Formamide, 0.3 μL Orange 500 size standard (Nimagen) and 1 μL undiluted PCR product. Controls were run on each plate to check for inter‐plate variation and scoring errors. Alleles were scored in Geneious Prime v2023.0.1 (Kearse et al. 2012). Four samples were excluded as more than two loci failed amplification, resulting in 1201 *B. pascuorum* and 1438 *B. terrestris* that were used in genetic analyses (Tables S3 and S4).

Samples were split into years and populations, and large islands from the 2022 data (Arran, Belle‐Ile, Guernsey, Isle of Man) were split into their five collection sites that were more than 3 km apart, so that foraging bees are unlikely to cross from one site into another. The 2022 Alderney samples were split into their two collection time points (June and August). Micro‐Checker v2.2.3 (Van Oosterhout et al. 2004) was used to check for null alleles and scoring errors by analysing each locus for homozygote excess. In *B. pascuorum*, B118 was excluded as a null allele in both years, B124 was excluded from the 2021 data, and B10 was removed from the 2022 data as it was not polymorphic or did not amplify in four populations (Table S5), resulting in 10 or nine loci (when using the combined dataset) that were used in the analysis. In *B. terrestris*, B119 was determined to be a null allele in 2021 but not in 2022 (Table S6), resulting in 11 and 12 loci in the analysis.

### 2.3 Colony Reconstruction

For bumble bee nests founded by a single monogamous queen, the number of nests in a given area corresponds to the effective population size (*N*_*e*_). *B. pascuorum* and *B. terrestris* colonies are founded by a usually singly mated queen (Estoup et al. 1995; Schmid‐Hempel and Schmid‐Hempel 2000), so we used the number of nests to determine *N*_*e*_. Sibship reconstruction was performed in COLONY v2.0.6.7 (Wang 2004), assuming male and female monogamy and using a medium length run with weak sibship prior. The maximum number of alleles available was used in this analysis: 10 loci for *B. pascuorum* in both years, but 12 for *B. terrestris* in 2022 and 11 in 2021. The inferred sibships with an estimated posterior probability of 0.8 or higher were accepted and used in downstream analyses (Dreier et al. 2014).

### 2.4 Population Structure

To remove possible confounding effects of family structure, we randomly chose one sample per colony before analysing the genetic structure. Using data from 2021 and 2022, we examined a total of 919 unique colonies in *B. pascuorum* and 1181 in *B. terrestris* (Tables S3 and S4). Analyses were conducted in R v4.2.2 (R Core Team 2014). Tests for departure from Hardy–Weinberg equilibrium were carried out using *adegenet* v2.1.10 (Jombart 2008) and corrected for multiple testing. Expected heterozygosity H_e_ (Nei 1978) was calculated using the package *poppr* v2.9.6 (Kamvar et al. 2014) and pairwise estimates of genetic diversity among sampling sites (*F*_ST_), inbreeding coefficients (*F*_IS_) and allelic richness (AR) averaged over all loci were calculated using the package *hierfstat* v5.511 (Goudet 2005). We used a Mantel test in *ade4* v1.7–22 (Dray and Dufour 2007) to test for correlation between pairwise *F*_ST_ and geographic distance (calculated using *geosphere* v1.5–18 (Hijmans et al. 2024)) with 999 permutations.

Using an Analysis of Molecular Variance (AMOVA), we looked for sources of genetic variation by partitioning variance among individuals, sampling years and sampling sites, using the package *poppr* v2.9.6 (Kamvar et al. 2014). Significant deviations from random population structures were tested using ‘randtest’ in *ade4* (Dray and Dufour 2007) with 999 bootstrap replicates. Significant and relatively high variance between populations could suggest population structuring. To analyse population structure and find clusters, we used two approaches (multivariate and Bayesian). Firstly, we performed a Discriminant Analysis of Principal Components (DAPC) using *adegenet* v2.1.10 (Jombart 2008). DAPC specifically seeks synthetic variables (the discriminant functions) that best reveal differences between groups while minimising variation within groups. We created ten subsamples of our data, limiting the number of individuals to 50 in each population and then used the function ‘xvalDapc’ to select the correct number of principal components with 100 replicates. Secondly, we used a Bayesian clustering algorithm in Structure v2.3.4 (Pritchard et al. 2000) using the admixture model, which assumes that individuals can have admixed recently. To account for uneven sampling sizes that could affect inference of population structure (Puechmaille 2016), we tested for structuring by randomly subsampling to 15 individuals per population ten times, using all sites and pooling both years. In some populations, fewer than 15 individuals were available, resulting in a total of 9 populations and *n* = 119 in *B. pascuorum* (Liverpool *n* = 6, Penryn *n* = 8) and 11 populations and *n* = 162 in *B. terrestris* (Liverpool *n* = 12). Models were run with 5000 burn‐in steps and 50,000 samples, with 10 iterations for each number of clusters (K), with K ranging from 1 to the number of populations plus 3. The optimal K was inferred by calculating the second‐order rate of change of the likelihood (∆K) (Evanno et al. 2005), and to detect fine scale structuring, we also observed K with the highest posterior probability as the log likelihood probability of the data (Pritchard et al. 2000) as implemented in the package *pophelper* v2.3.1 (Francis 2017). We then corrected this optimal K by removing clusters with a mean membership < 0.5 in any population, as those ‘ghost clusters’ can be considered spurious (Puechmaille 2016). For each population, the mean cluster membership across all subsets was used for plotting, using K with the highest corrected posterior probability.

### 2.5 Colony Density

For the following analyses, we focused on the data set from 2022 (22 sites with *n* = 985 *B. pascuorum* and 21 sites with *n* = 892 *B. terrestris* ), which covered fewer populations but offered higher replication within the six island populations. To estimate the effective population sizes, the number of colonies was estimated by inferring sister pairs as capturing the same colony multiple times in genetic mark‐recapture models (Miller et al. 2005). Population size was estimated by fitting the equal capture model (ECM) and the two innate rates model (TIRM) with a maximum population size of 1000 colonies using the package *capwire* v1.1.4 (Pennell et al. 2013) in R v4.0.5 (R Core Team 2014). A likelihood ratio test was used to compare model fits using 100 bootstraps. Confidence intervals for the population size estimates were calculated by performing 100 parametric bootstraps. No siblings were found for *B. terrestris* in Lamlash (Arran) and Pierre du Bois (Guernsey), so the population size could not be estimated. Only one *B. terrestris* sister pair was found in Lagg (Arran) and the Dalby Mountain reserve (Isle of Man), resulting in estimates of population size with large errors that were not used for colony density calculations.

Colony density was then estimated for 22 sites in *B. pascuorum* and 17 in *B. terrestris* using the number of colonies and sampling area. Note that for Alderney, which was sampled in June and August, population size and colony density were calculated for both months separately. Samples collected on large islands were collected within a 500 m radius at each site. Yet, the true area sampled is affected by the foraging range of the bumble bee species, as individuals from nests outside the 500 m radius can forage within the sampling site. We therefore added a foraging range of 601 m for *B. pascuorum* (Carvell et al. 2012; Knight et al. 2005; Wood et al. 2015) and 1040 m for *B. terrestris* (Osborne et al. 1999, 2008; Walther‐Hellwig and Frankl 2000; Wolf and Moritz 2008; Wood et al. 2015) to the 500 m radius to calculate the area sampled (3.81 km^2^ and 7.45 km^2^, respectively). On Alderney and Ouessant, our search covered the whole island (6.8 km^2^ and 15.6 km^2^, respectively).

### 2.6 Pathogen Prevalence

To study how population structure affects pathogen prevalence, we used island samples from 2022 (*n* = 889 *B. pascuorum* and *n* = 803 *B. terrestris* ), as they provide finite populations that can be clearly separated by location. Samples collected on Alderney in June (49 *B. pascuorum* and 62 *B. terrestris* ) and some collected from Guernsey (47 *B. pascuorum* and 27 *B. terrestris* ) had to be excluded from the pathogen analysis as samples had defrosted during transport (Tables S3 and S4) and were therefore not available for pathogen screening. We screened bumble bees for nine pathogens. Tissue from half bees (laterally bisected) was homogenised (speed 5 m/s for 25 s with 3 cycles and 20 s pause using a FisherBrand Bead Mill 24), and RNA was extracted using 1.3 mL TRI‐Reagent (Sigma‐Aldrich) and 0.1 mL Bromo‐Chloro‐Propanol (Sigma‐Aldrich). RNA was eluted in 100 or 120 μL H_2_O, for *B. pascuorum* and *B. terrestris* , respectively, and concentrations were measured by fluorescent dye (QuantiFluor RNA System, Promega).

600–3750 ng RNA was reverse transcribed using random hexamers and GoScript reverse transcriptase (Promega) according to the manufacturer's instructions to screen for four RNA viruses (black queen cell virus (BQCV), deformed wing virus type A and B (DWV‐A and DWV‐B), slow bee paralysis virus (SBPV)) and for mRNA from five parasites (*Apicistis bombi*, *Crithidia bombi*, *Nosema apis*, *N. bombi* and *N. ceranae*). PCR was performed using the GoTaq Flexi kit (Promega) (details in Tables S7 and S8). Every run included a known virus‐positive sample and water as a negative control. 5 μL PCR product was visualised by 1.5% (2.5% for *Nosema* spp.) TAE agarose gel electrophoresis and staining with RedSafe (Intron Biotechnology) using HyperLadder 50 bp (Bioline) to check the fragment size.

### 2.7 Effects of Population Structure on Pathogen Prevalence

True pathogen prevalence at each sampling site and on each island was calculated using the package *epiR v2.0.75* (Stevenson et al. 2013) with 98% sensitivity (true positive rate) and 98% specificity (true negative rate) (Reiczigel et al. 2010). DWV‐A and *N. apis* were not detected, and BQCV was only detected in five individuals and excluded from further analyses. To test if pathogen prevalence was affected by colony density and genetic diversity, we ran generalised linear mixed models (GLMMs) with binomial distribution and logit link function using the package *lme4* v1.1–34 (Bates et al. 2015). Model selection was done by fitting the full model with colony density, expected heterozygosity (H_e_), latitude (as a proxy for distance from glacial refugia), *Varroa destructor* (varroa) presence and prevalence of other pathogens as predictors, island as a random effect and removing non‐significant covariates with the largest corresponding *p*‐value (*α* = 0.05). Varroa is a parasitic mite in honey bees that vectors viruses and has been linked to increased spillovers into bumble bee populations. In 2021 and 2022, this vector was absent on Alderney, the Isle of Man and the Scilly Isles, but present at all other sites (Dobelmann et al. 2024; Manley et al. 2019). Allelic richness (AR) was excluded from model selection, as it is strongly affected by sampling size and showed a strong correlation with H_e_. We note that with our sampling design, latitude was correlated with sampling date (Pearson's correlation *r* = 0.685, df = 20, *p* < 0.001), so that geographical variation may also be linked to seasonal differences. We used the *anova*() function to test whether including the random effect improved the model fit and used generalised linear models (GLMs) when the random effect could be omitted. To test if any parameters showed infinite maximum likelihood estimates that can indicate quasi‐complete separation, we used *detectseparation* v0.3 (Kosmidis et al. 2022) and confirmed that no model estimator was infinite. Fitted models were tested for over‐dispersion and zero inflation using the package *DHARMa* v0.4.6 (Hartig 2020), showing no significant over‐dispersion or zero inflation. We also checked for collinearity using the package *car* v3.1–3. To correct for multiple testing, we used Benjamini‐Hochberg correction (*Q* = 0.05). None of the significant results were rejected, so we report unadjusted *p*‐values.

### 2.8 Pathogen Dissimilarity in Sisters and Unrelated Individuals

Presence‐absence data from the five most common pathogens found in bees were used to calculate pairwise pathogen dissimilarities (Hamming distances) between bees collected within the same site. We used 275 and 185 pairwise comparisons of sisters and 18,811 and 16,070 pairwise comparisons of unrelated individuals within 22 sites for *B. pascuorum* and 17 sites for *B. terrestris* , respectively. *B. terrestris* samples from four sites with no sisters or only one sister pair were excluded from the analysis. Next, we modelled the probability of higher dissimilarity scores as a function of sisters vs. non‐sisters. Distances ranged from 0 to 5 and were used in an ordinal regression with cumulative link mixed models (CLMM) with a logit link using the package *ordinal* v2023.4.1 (Christensen 2018). CLMMs have been shown to robustly handle uneven sampling sizes (Taylor et al. 2023), i.e., fewer observations for sisters than non‐sisters. Pathogen dissimilarity was used as the ordinal response, pairing (sisters vs. non‐sisters) as a fixed effect, and IDs from the two individuals in each pairwise comparison were used as random effects to account for non‐independence. We tested whether pairing as a predictor improved model fit using a likelihood ratio test comparing the fitted model to a null model. For the five individual pathogens, GLMMs with binomial response with logit link function and individual IDs as random effects were used. To correct for multiple testing, significance thresholds were adjusted using the Benjamini–Hochberg correction (*Q* = 0.05).

## 3 Results

### 3.1 Population Structure

Genetic distances between mainland sites were low (*F*_ST_ < 0.05), but in *B. pascuorum* , all islands, except for Arran, showed moderate (0.05 < *F*_ST_ < 0.15) distance to the mainland (Table S9). Differentiation between *B. pascuorum* island populations was moderate to high, with the smallest *F*_ST_ between the Isle of Man and Arran (*F*_ST_ = 0.074) and the largest between Guernsey and Ouessant (*F*_ST_ = 0.285). A significant amount of genetic variation could be attributed to differences between populations (AMOVA: 25.82%, *p* < 0.001), with a weak difference between sampling years within populations (0.70%, *p* = 0.043), but most variation was found within populations (74.18%, *p* < 0.001). We found no correlation between geographic and genetic distance in *B. pascuorum* (Mantel test: *r* = −0.060, *p* = 0.608, Figure S2). The population structure analysis using DAPC showed a central cluster including Arran and all mainland sites, from which the Isle of Man, the Channel Islands (Alderney and Guernsey), Ouessant and Belle‐Ile separated into distinct clusters with increasing separation from this central cluster (Figure S3). This pattern was consistent across all subsampled data sets (Figure S3). Further assessing the genetic patterns using Structure, we found that optimal K ranged from two to five clusters using ∆K (median *K* = 2) and three to five K when using the highest posterior probability (median *K* = 5, Table S10). The interpretation over increasing K, however, was consistent across subsets: With two clusters (*K* = 2), some individuals from Ouessant formed a separate cluster, but with three clusters (*K* = 3), the Channel Islands (Alderney and Guernsey) separated from the other populations. Introducing a fourth cluster (*K* = 4) separated Belle‐Ile and a fifth (*K* = 5) separated the Isle of Man from the large central cluster (Figure 1, Figure S4). The Channel Islands split into two clusters at *K* = 7 or *K* = 8, but further increasing K did not lead to a separation of the mainland sites (Liverpool, Penryn and Le Conquet) nor a separation of Arran from these mainland sites, even though Arran is an island and Le Conquet on the French mainland is separated from the British sites by the English Channel.

In *B. terrestris*, genetic distances between populations were overall smaller (*F*_ST_ ≤ 0.054), except for the Isle of Man and the Scilly Isles, which showed moderate differentiation from all other sites (0.05 < *F*_ST_ < 0.15) and high differentiation from each other (*F*_ST_ = 0.190, Table S9). Still, a significant amount of genetic variation could be attributed to differences between populations (AMOVA: 9.58%, *p* < 0.001), with fewer differences between years within populations (2.78%, *p* < 0.001). As for *B. pascuorum* , most of the variation was found within the populations (87.64%, *p* < 0.001). We found no significant relationship between genetic distance and geographic distance in *B. terrestris* (Mantel test: *r* = 0.204, *p* = 0.177); yet, when we excluded the two populations that were genetically differentiated from the majority of sites (the Isle of Man and the Scilly Isles), genetic distance significantly correlated with geographic distance (*r* = 0.620, *p* = 0.001, Figure S2). Most populations in the DAPC overlapped without forming distinct genetic clusters, except for the Isle of Man and the Scilly Isles (Figure S5). The remaining sites split into a British and French (plus Channel Islands) group, with a large overlap within but only some overlap between groups. This pattern occurred in all subsampled data sets. *B. terrestris* showed weaker population structuring than *B. pascuorum*, with the highest ΔK ranging from two to three clusters (median *K* = 2) and the highest posterior probability between three to four clusters (median *K* = 4, Table S11). Again, the clustering over increasing K was consistent across subsets. At *K* = 2, the British Isles separated from the Channel Islands and France. Introducing a third cluster (*K* = 3) separated the Scilly Isles and a fourth cluster (*K* = 4) separated the Isle of Man from the other populations on the British Isles (Figure 1, Figure S6). However, at *K* = 4, Arran and, to a lesser extent, Liverpool, also had high membership probabilities to the Isle of Man cluster, but a fifth (*K* = 5) or sixth (*K* = 6) cluster always confirmed a separation of the Isle of Man from other British sites (Figure S6). Introducing more clusters did not yield separation within the remaining British group (Arran, Liverpool, Penryn) or the group that included France (Belle‐Ile, Le Conquet, Ouessant, Quiberon) and the Channel Islands (Alderney and Guernsey). For both bee species, no genetic differentiation (*F*_ST_ > 0.05) was found between samples collected from different sites within the same island.

### 3.2 Bumble Bee Colony Density and Genetic Diversity

**FIGURE 2:**
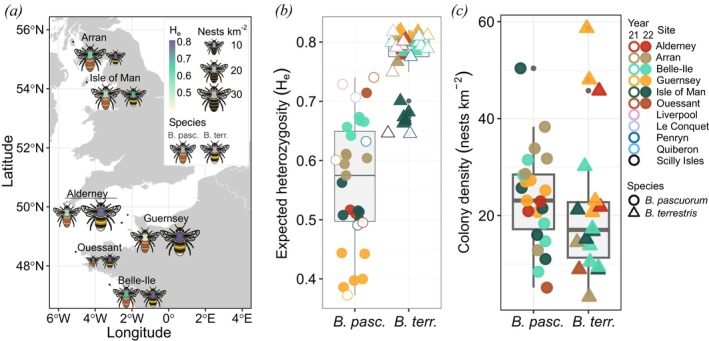
(a) The map shows the average expected heterozygosity (H_e_) and colony density of *Bombus pascuorum* (left) and *B. terrestris* (right) nests in six island populations. The thorax colour of the bee shows the average expected heterozygosity (H_e_) and the size is scaled by the average colony density (nests km^−2^). Boxplots show (b) the expected heterozygosity (H_e_) and (c) the colony density (nests km^−2^) in *B. pascuorum* (circles) and *B. terrestris* (triangles). Shapes are coloured by population and represent different sites for island samples collected in 2022 (filled shapes) in (b) and (c). In (b), H_e_ is also shown for mainland (Liverpool, Le Conquet, Penryn, Quiberon) and island (Alderney, Arran, Belle‐Ile, Guernsey, Isle of Man, Ouessant, Scilly Isles) samples collected in 2021 (shapes with white fill).

Colonies were on average represented by 1.307 workers in *B. pascuorum* and 1.217 workers in *B. terrestris* . No deviation from Hardy–Weinberg equilibrium was found. Genetic diversity (H_e_) was smaller in *B. pascuorum* (mean = 0.568 ± 0.019) and spanned a wider range compared to *B. terrestris* (mean = 0.766 ± 0.001, t‐test: *p* < 0.001, Figure 2a). Within species and islands, H_e_ was similar at all sampling sites but differed between islands (Tables S3 and S4). For *B. pascuorum* , Ouessant had the highest diversity (H_e_ = 0.727) out of all islands, similar to the heterozygosity found in mainland sites (Figure 2b, Table S3). Interestingly, the lowest H_e_ in this species was found on Guernsey (H_e_ = 0.407), while in *B. terrestris*, this island showed the highest heterozygosity (H_e_ = 0.814) out of the seven islands (Figure 2b, Table S3). H_e_ in *B. pascuorum* from islands was negatively correlated with the island's distance from the mainland (Pearson's correlation *R* = −0.866, *p* < 0.001, Figure S7), while in *B. terrestris*, diversity was mostly affected by latitude, with lower diversity in its northern range (Pearson's correlation *R* = −0.655, *p* = 0.004, Figure S7). Most islands showed similar H_e_ in *B. terrestris* except for the Isle of Man and the Scilly Isles, which had the lowest diversity (H_e_ = 0.672 and H_e_ = 0.646, respectively, Figure 2b). Differences between island and mainland sites were small. On average, *B. pascuorum* on islands had a slightly lower H_e_ compared to the mainland (0.555 and 0.689, respectively, Figure 2b, Table S3). In *B. terrestris*, this difference was weaker (H_e_ = 0.769 on islands and H_e_ = 0.800 on the mainland, Table S4).

Island samples from 2022 were used to estimate colony density, which did not differ between species (*t*‐test: *p* = 0.617). In *B. pascuorum* , we found densities ranging from 5 colonies km^−2^ on Ouessant to 50 colonies km^−2^ at Dalby Mountain reserve on the Isle of Man (mean = 23.6 ± 2.12, Figure 2c, Table S3). In *B. terrestris* , densities ranged from 3 colonies km^−2^ in Brodick on Arran to 59 colonies km^−2^ in Saumarez Park on Guernsey (mean = 21.6 ± 3.21, Figure 2c, Table S4). The colony density for *B. terrestris* increased with the islands' distance from the mainland (Pearson's correlation *R* = 0.618, *p* = 0.008, Figure S7). Both islands with low colony density showed moderate inbreeding ( *B. pascuorum* on Ouessant: *F*_IS_ = 0.198, Table S3, and *B. terrestris* on Arran: *F*_IS_ = 0.104, Table S4), while all other populations had low inbreeding coefficients (*F*_IS_ < 0.1). We found no correlation between colony density and H_e_ (Pearson's correlation: *B. pascuorum* *R* = −0.203, *p* = 0.352 and *B. terrestris* *R* = 0.261, *p* = 0.296), but allelic richness and H_e_ showed a strong correlation ( *B. pascuorum* : *R* = 0.824 and *B. terrestris* : *R* = 0.973, both *p* < 0.001, Figure S8).

### 3.3 Pathogen Prevalence

**FIGURE 3:**
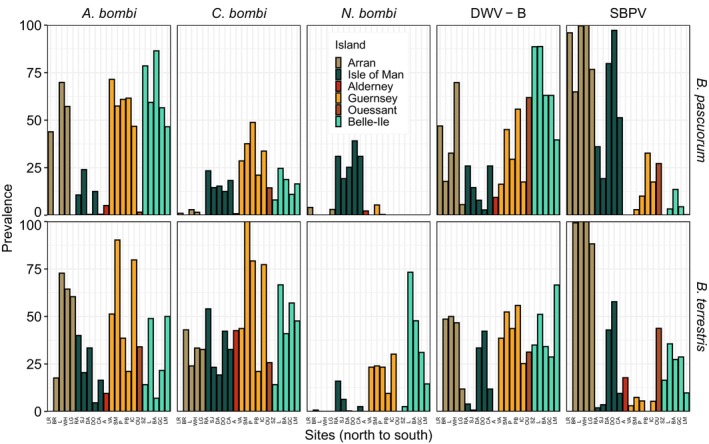
True prevalence of five pathogens (*Apicystis bombi*, *Crithidia bombi*, *Nosema bombi*), deformed wing virus type B (DWV‐B) and slow bee paralysis virus (SBPV) in *Bombus pascuorum* (top) and *B. terrestris* (bottom) at 22 collection sites on six islands (sorted north to south). Note that only one *B. terrestris* was found in the northernmost site and excluded from the analysis. Alderney and the Isle of Man are free of the honey bee virus vector *Varroa*.

Bumble bees were tested for nine pathogens on island populations in 2022 to assess how infections are linked to population structure. *A. bombi* and *C. bombi* infections were found in both bee species and on all islands, although both pathogens were absent at two sites on Arran in *B. pascuorum* (Figure 3). In this bee species, prevalences ranged from 0% to 86% for *A. bombi* and 0% to 49% for *C. bombi*. In *B. terrestris* , prevalence ranged from 5% to 90% for *A. bombi* and from 14% to 100% for *C. bombi*. The gut parasite *Nosema* spp. showed large differences in prevalence between islands. *N. apis* was absent from all islands, and *N. ceranae* was only found in one *B. terrestris* from Lagg on Arran and 39 *B. pascuorum*, mostly from Belle‐Ile (34) and some from Arran (5), with the highest prevalence in Bangor on Belle‐Ile (34%) and hence excluded from further analyses. *N. bombi* was the most common out of the three *Nosema* spp. screened for, but absent on Ouessant and rare on Alderney (2% in *B. pascuorum* and no detection in *B. terrestris* ) and Arran (up to 4% in *B. pascuorum* and 1% in *B. terrestris*, Figure 3). *N. bombi* infections were more common on the Isle of Man, where mostly *B. pascuorum* tested positive, and Belle‐Ile and Guernsey, where *B. terrestris* tested positive (Figure 3). Interestingly, on Belle‐Ile, *N. ceranae* was dominant in *B. pascuorum* , but *N. bombi* in *B. terrestris* . The viral pathogens SBPV and DWV‐B were detected on all islands and generally in both bee species, but on Alderney, the smallest island, SBPV was absent in *B. pascuorum* and DWV‐B in *B. terrestris* (Figure 3). Prevalence for SBPV was highest on Arran (up to 100% in both species) and for DWV‐B on Belle‐Ile (up to 89% and 67%, in *B. pascuorum* and *B. terrestris*, respectively). DWV‐A was not detected, and BQCV was rare and excluded from further analyses.

### 3.4 Effects of Population Structure on Pathogen Prevalence

**FIGURE 4:**
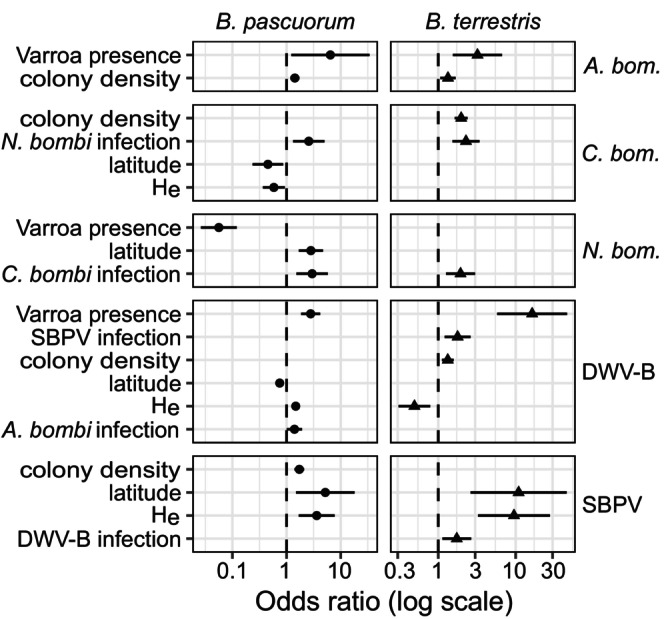
Summary plot showing odds ratios with 95% confidence intervals on a log scale for fitted generalised linear models and generalised linear mixed models explaining pathogen prevalence in *Bombus pascuorum* (circles) and *B. terrestris* (triangles). The random term ‘island’ was removed when it was not significantly different from zero, so that for *Crithidia bombi* in *B. terrestris* , *Nosema bombi* in *B. pascuorum* and deformed wing virus B (DWV‐B), generalised linear models were used. A positive odds ratio indicates a term that increases the likelihood of an *Apicystis bombi* (*A. bom*.), *C. bombi* (*C. bom*.), *N. bombi* (*N. bom*.), DWV‐B, or slow bee paralysis virus (SBPV) infection. Predictors were colony density (nests km^−2^), expected heterozygosity (H_e_), presence of varroa on the island, latitude and co‐infections with other pathogens. After fitting a full model, non‐significant predictors were removed.

Linear models to describe pathogen prevalences showed that island as a random effect did not improve the model fit for *C. bombi* in *B. terrestris* , *N. bombi* in *B. pascuorum* and DWV‐B in either species and was hence removed. Large variability in the random effect in both SBPV models (random intercept up to ±2.8) could suggest that factors not included here affect SBPV prevalence or that the model struggles to estimate the very high prevalences in some sites and low prevalences in others. Colony density increased the odds of *A. bombi* infections in both species (Figure 4, Table S12). Other pathogens were also increased in denser populations (*C. bombi* and DWV in *B. terrestris* and SBPV in *B. pascuorum* (Figure 4)). Contrary to expectations, genetic diversity (H_e_) increased the likelihood of SBPV infections in both species and DWV‐B infections in *B. pascuorum* , although in *B. terrestris* , DWV‐B decreased with higher diversity and in *B. pascuorum, C. bombi* infections also decreased with H_e_. The presence of the honey bee parasite varroa increased the odds of detecting *A. bombi* and DWV‐B in both species but decreased *N. bombi* in *B. pascuorum* (Figure 4). However, as the varroa‐free Isle of Man was the only island with < 10% prevalence (Figure 3), this may have driven the negative effect in *B. pascuorum* . Additionally, individuals from varroa‐present islands were on average sampled 30 days earlier in the year, so that seasonal variation cannot be separated from this varroa effect. To account for geographical variation, we also included latitude in the model selection. Higher latitudes had higher odds of SBPV infections, while for other pathogens, the effects were weaker and mixed (Figure 4). Infections with *N. bombi* increased the odds of *C. bombi* infection and vice versa, and the two viruses also increased each other's prevalence in *B. terrestris* (Figure 4).

### 3.5 Pathogen Dissimilarity in Sisters and Unrelated Individuals

Overall, differences in pairwise pathogen infection profiles between nestmates and unrelated individuals were small in both *B. pascuorum* (mean pathogen dissimilarity ± standard error: 1.17 ± 0.19 vs. 1.23 ± 0.18, respectively) and *B. terrestris* (1.50 ± 0.14 vs. 1.58 ± 0.14, respectively). In *B. pascuorum* , sisters were on average 0.225 units lower on the dissimilarity scale (CLMM Table S13), but including pairing did not significantly improve the fit over the null model (likelihood ratio test: χ² = 2.94, df = 1, *p* = 0.086) and no individual pathogen showed more similar pathogen profiles between sisters (Table S14). In *B. terrestris* , however, the odds of having more similar pathogen profiles were significantly higher for sisters (CLMM: *β* = −0.453, SE = 0.154, *p* = 0.003, Table S13). This effect was driven by a 1.6 times higher probability of sisters having the same *C. bombi* infection status (*p* = 0.009) and a 3.5 times higher probability of the same *N. bombi* infection status (*p* < 0.001) than individuals from different nests (Table S15).

## 4 Discussion

Here, we examined the population structure of *B. pascuorum* and *B. terrestris* in multiple island and mainland sites in the British Isles and France and then linked pathogen prevalence to this population structure and, within populations, to nest membership. Islands created barriers for gene flow, causing structuring particularly in *B. pascuorum* . At the population level, greater colony density had a slight but always increasing effect on pathogen prevalence, while genetic diversity had no general protective effect and was instead linked to increased viral prevalence. Within populations, *B. terrestris* nestmates shared similar pathogen profiles; however, the mean pathogen similarity in nestmates did not differ much from unrelated individuals.

### 4.1 Islands Act as Barriers to Gene Flow in Bumble Bees

We found population structuring in both *B. pascuorum* and *B. terrestris*, two species that generally show little population structure on the European continent (Moreira et al. 2015; Widmer and Schmid‐Hempel 1999). *B. terrestris* showed less population structuring compared to *B. pascuorum*, with the cluster analysis separating *B. terrestris* into its subspecies, *B. t. audax* in the British Isles and *B. t. terrestris* in France and on the Channel Islands (Lecocq et al. 2016). However, clusters varied gradually from Arran in the north to Belle‐Ile in the south and geographic distance accounted for genetic distance when we removed the two genetically differentiated islands, the Isle of Man and Scilly Isles. This suggests that a high connectivity between island and mainland populations can allow for stepwise accumulation of genetic differences in *B. terrestris* . In contrast, *B. pascuorum* showed stronger genetic clustering, with all islands except for Arran and some individuals from Ouessant being genetically differentiated from the mainland and forming separate genetic clusters. Low connectivity between island and mainland sites in *B. pascuorum* could indicate isolation by resistance (McRae 2006), which, in addition to geographic distance, acknowledges habitat heterogeneity such as the water surrounding the islands. Limited long‐distance migration with an average queen dispersal of only 1.3 km (Lepais et al. 2010) could explain why Arran, which is located 5 km off the Scottish west coast, but not other islands, which are located at least 10 km from the nearest mainland, clusters with the mainland population. However, we didn't find separation between *B. pascuorum* at mainland sites north and south of the English Channel that are separated by at least 32 km of water and show phenotypic variation in colour patterns (Lecocq et al. 2015). In comparison, *B. terrestris* queens disperse 3 km on average (Makinson et al. 2019), but invasive populations show even greater long‐distance migration of 25–100 km per year (Kingston et al. 2002; Morales et al. 2013). In addition, accidental or intentional human‐mediated movement may be more common in *B. terrestris* . The below‐ground nesting species could accidentally be translocated with soil, which appears less likely for *B. pascuorum* that nests above or close to the ground (Fussell and Corbet 1992). Moreover, commercially reared *B. terrestris* are traded for greenhouse pollination, and even though little introgression of commercial bees into UK populations has been detected so far (Franchini et al. 2023; Hart et al. 2021), such movement can increase connectivity and obscure natural isolation patterns or potentially disrupt locally adapted gene complexes. Taken together, water can be a strong barrier for dispersal (Liu et al. 2024) but its effects vary between bumble bee species.

### 4.2 Does Genetic Diversity Affect Pathogen Prevalence?

Population structuring and dispersal barriers can affect how genetic diversity is distributed. Overall, *B. terrestris* showed higher genetic diversity (H_e_) compared to *B. pascuorum*, a pattern consistent with observations in Germany and the UK (Dreier et al. 2014; Parsche and Lattorff 2018), so that this difference in genetic diversity may not arise from sampling island locations. However, in *B. pascuorum*, island distance from the mainland negatively correlated with genetic diversity, and some islands showed reduced genetic diversity in comparison to mainland sites. In *B. terrestris* , genetic diversity decreased with latitude, which could be caused by recolonization of previously glaciated regions, northward from southern refugia (Kelemen and Rehan 2021; Ranjbaran et al. 2024) and fits with the general pattern of peripheral populations exhibiting lower diversity found in many species (Eckert et al. 2008). It should be noted that the two genetically separated islands, the Isle of Man and the Scilly Isles, had low genetic diversity and were among the northernmost locations, potentially contributing to this effect. The overall greater degree of population structuring and lower genetic diversity in *B. pascuorum* compared to *B. terrestris* could possibly make *B. pascuorum* more susceptible to population isolation or create conditions that favour pathogen transmission.

We hypothesised that populations with low genetic diversity would facilitate pathogen transmission and lead to higher pathogen prevalence. Contrary to our expectation, more genetically diverse populations showed no reduction in pathogen prevalence. For viral pathogens, increased heterozygosity was even associated with an increase in prevalence. However, the pathogens and parasites investigated here all show a broad host spectrum within bumble bees and beyond and are widely transmitted within the pollinator community (Burnham et al. 2021; Fürst et al. 2014; Graystock et al. 2015). For such multi‐host pathogens, biodiversity at the host level could be a better predictor for pathogen prevalence than genetic diversity at the species level. Indeed, pollinator‐friendly conservation schemes that increase bumble bee diversity and density (Doublet et al. 2022; Wood et al. 2015) can reduce viral prevalence via a dilution effect (Fearon and Tibbetts 2021; Manley et al. 2023; Maurer et al. 2024). This dilution effect may differ for different types of pathogens; for instance, increased exposure to other pollinators through the establishment of wildflower fields in poor quality landscapes can enhance micro‐parasite prevalence in wild bumble bees and even create transmission hot spots (Piot et al. 2019). Contrasting multi‐host pathogens with specialist pathogens could resolve these sometimes conflicting patterns and disentangle the importance of biodiversity and genetic diversity for disease prevalence in natural populations.

### 4.3 Is Host Density Driving Pathogen Prevalence?

Since many bee pathogens are faecal‐orally transmitted (Figueroa et al. 2019), their transmission is expected to be density‐dependent (Anderson and May 1979), so that we expected higher prevalences in denser populations. Overall, we find similar colony densities for both species in the seminatural and suburban habitats sampled here. Agricultural habitats, in contrast, typically have higher *B. pascuorum* than *B. terrestris* densities (Darvill et al. 2004; Knight et al. 2005). Bee densities and floral resources can be influenced by human activity, such as gardens and agricultural fields, which affect pathogen dynamics (Becker et al. 2015). Therefore, suburban habitats, which can support a high density of social bees, have been linked to increased prevalence of *A. bombi*, *C. bombi* and *N. bombi* when compared to rural areas with lower density (Goulson et al. 2012; Theodorou et al. 2016). We find that colony density increased the prevalence of some micro‐parasites (*A. bombi* and *C. bombi*) and viruses (DWV‐B and SBPV), but not always in both hosts. Inferred colony density may not always reflect true densities, as foraging ranges are affected by floral communities (Jha and Kremen 2013) and mass flowering of crops, for instance, increases nest size rather than nest density (Herrmann et al. 2007). Still, most variation in colony density was found between islands, not between species, so that colony density could indicate resource availability that may affect the density of multiple host species.

*B. terrestris* and *B. pascuorum* are among the most common bumble bees found in the UK (Whitehorn et al. 2022), yet, on the Scottish Isle of Arran, colony density was high for *B. pascuorum* but surprisingly low for *B. terrestris* and we detected many *B. lucorum* in our initial screening. While *B. lucorum* prefers wet and cold habitats with dense vegetation (Geue and Thomassen 2020), such as are found in Scotland, at similar latitudes in Sweden, *B. terrestris* prevalence is increasing with warming climates, possibly leading to a replacement of *B. lucorum* (Herbertsson et al. 2021). The low *B. terrestris* colony density on Arran may hence reflect a different bumble bee species composition. Interestingly, latitude had a strong positive effect on SBPV prevalence, which reached up to 100% in both species on Arran. While temperature and precipitation extremes have been linked to lower rather than higher viral prevalences (Piot et al. 2022), perhaps a different bumble bee community composition or the colder climate facilitated SBPV transmission. Yet, latitude positively correlated with sampling date so that sampling later in the season could also affect viral prevalence in bees (D'Alvise et al. 2019).

In addition to wild bees, managed bees in the pollinator community can affect bumble bee densities and pathogen infections. On Ouessant, for instance, both bumble bee species nested at low densities (< 10 nests km^−2^), possibly due to competition with honey bees, which are kept at high densities of around 17.8 colonies km^−2^ on the island (Ménage and L'Hostis 2021). Such high honey bee abundance is known to decrease bumble bee abundance and species diversity (MacInnis et al. 2023; Su et al. 2022). Since viral prevalences in wild bees strongly correlate with those found in managed honey bees (Maurer et al. 2024; Piot et al. 2022), this low bumble bee density may not link to lower pathogen prevalence. Instead, vector transmission via varroa that has been introduced in honey bee populations on Arran, Belle‐Ile, Guernsey and Ouessant is linked to a DWV spillover resulting in increased prevalence and loads in bumble bees (Dobelmann et al. 2024; Fürst et al. 2014; Manley et al. 2020, 2019). In addition to DWV‐B, we found that the presence of varroa was associated with increased *A. bombi* prevalence. This emerging micro‐parasite is rarely found in *Apis mellifera* (Plischuk et al. 2011), so perhaps the geographic isolation and strict biosecurity of some varroa‐free islands also reduce the import of pathogens from mainland bumble bee populations or commercially reared bumble bees (Murray et al. 2013). Spillover of *A. bombi* and C. *bombi* has been detected from greenhouses with commercially reared bumble bees into wild bumble bee populations (Graystock et al. 2014; Otterstatter and Thomson 2008). These multi‐host pathogen dynamics suggest that colony density of one species alone may not be a good predictor for multi‐host pathogen prevalence.

### 4.4 Does Nest Membership Affect Pathogen Profiles?

Close interactions within the nest and genetic similarity can favour pathogen transmission (Schmid‐Hempel 1998). Indeed, we find that *B. terrestris* sisters were more likely to have similar pathogen profiles than individuals from different nests. When looking at individual pathogens, nestmates were more likely to have the same *N. bombi* and *C. bombi* infection status. Higher transmission rates within social groups are well documented in wild‐living mammals (Altizer et al. 2003; Drewe 2010), but less so in insects. An increased transmission within bumble bee colonies, due to their genetic similarity, has been shown for *C. bombi* (Baer and Schmid‐Hempel 1999), where parasite genotype by host genotype interactions affect parasite infectivity and host susceptibility (Schmid‐Hempel and Ebert 2003). Mitochondrial DNA haplotype has been linked to *N. bombi* prevalence, suggesting interactions with the host genotype (Manlik et al. 2017). Yet, in wild populations, interactions with co‐infecting pathogens not measured here or differences in foraging patterns between nests may also affect infection outcomes, so that the inter‐colony transmission is just one of the mechanisms by which nestmates may share more similar pathogen profiles.

## 5 Conclusion

Island populations of *B. pascuorum* and, to a lesser extent, *B. terrestris* show population structuring. While population structure can affect pathogen prevalence, we find no evidence for low genetic diversity populations with high density suffering from increased pathogen prevalences. Instead, complex interactions between co‐occurring pathogens, hosts, their population structure and environmental factors affect pathogen occurrence. Yet, within populations, we demonstrate that *B. terrestris* nestmates are more similar in their pathogen infection profiles than unrelated individuals, indicating that the nest environment may significantly contribute to pathogen transmission in wild populations. Even though population genetic diversity did not reduce pathogen infections, it is important for population resilience. With the increasing pressures of habitat change, agricultural intensification and the spread of managed pollinators, island populations with restricted gene flow and low genetic diversity may be particularly vulnerable to environmental pressures including pathogens. Understanding the dynamics of population structure and pathogen transmission can aid the conservation of genetically isolated populations and improve the health of pollinator communities.

## Author Contributions

The study was designed by L.W. and J.D. L.W. acquired funding. J.D. performed field and laboratory research, with contributions from L.W. J.D. analysed the data with input from L.W. The paper was written by J.D. with input from L.W.

## Disclosure

Benefit Sharing: Benefits from this research accrue from the sharing of our data and results on public databases as described above.

## Conflicts of Interest

The authors declare no conflicts of interest.

## Supporting information


**Appendix S1:** PCR to distinguish between *Bombus terrestris* and *B. lucorum*.
**Appendix S2:** Microsatellite PCRs.
**Table S1:** Locations and sampling time points for *Bombus pascuorum* and *B. terrestris* collections in 2021 and 2022.
**Table S2:** Primers used for microsatellite amplification.
**Table S3:** Summary of genotyped workers, sibship reconstructions, colony density and genetic diversity in *Bombus pascuorum*.
**Table S4:** Summary of genotyped workers, sibship reconstructions, colony density and genetic diversity in *Bombus terrestris*.
**Table S5:** Characteristics of 12 microsatellite loci in *Bombus pascuorum*.
**Table S6:** Characteristics of 12 microsatellite loci in *Bombus terrestris*.
**Table S7:** PCR conditions used to screen for pathogens.
**Table S8:** Primer sequences used for pathogen screening.
**Table S9:** Pairwise F_ST_ between populations from island and mainland sites in *Bombus pascuorum* populations and *B. terrestris*.
**Table S10:** Summary statistics from STRUCTURE runs using 10 subsets of the data with 119 individuals genotyped at 9 loci in *Bombus pascuorum*.
**Table S11:** Summary statistics from STRUCTURE runs using 10 subsets of the data with 162 individuals genotyped at 10 loci in *Bombus terrestris*.
**Table S12:** Best generalised linear mixed models or generalised linear models explaining pathogen prevalence.
**Table S13:** Cumulative link mixed models to test whether nest membership affects pathogen dissimilarity.
**Table S14:** Generalised linear mixed models to test whether nest membership affects individual pathogen presence in *Bombus pascuorum*.
**Table S15:** Generalised linear mixed models to test whether nest membership affects individual pathogen presence in *Bombus terrestris*.
**Figure S1:** Maps showing sampling sites.
**Figure S2:** Genetic distance by geographic distance between the populations of *Bombus pascuorum* and *B. terrestris*.
**Figure S3:** Scatterplots from ten random subsampled data sets showing the discriminant analysis of principal components (DAPC) of the first two principal components discriminating *Bombus pascuorum* populations.
**Figure S4:** Proportional membership of *Bombus pascuorum* island and mainland samples from 2021 and 2022 from K = 2 to K = 12 genetic clusters.
**Figure S5:** Scatterplots from ten random subsampled data sets showing the discriminant analysis of principal components (DAPC) of the first two principal components discriminating *Bombus terrestris* populations.
**Figure S6:** Proportional membership of *Bombus terrestris* island and mainland samples from 2021 and 2022 from K = 2 to K = 14 genetic clusters.
**Figure S7:** Scatterplot showing the correlation between *(a)* island distance from the mainland and expected heterozygosity (H_e_), *(b)* island distance from the mainland and colony density or *(c)* latitude and expected heterozygosity (H_e_) in *Bombus pascuorum* and *B. terrestris*.
**Figure S8:** Scatterplot showing a strong correlation between *(a)* allelic richness (AR) and expected heterozygosity (H_e_) and between *(b)* colony density and H_e_ in *Bombus pascuorum* and *B. terrestris*.

## Data Availability

Individual allele data and pathogen prevalence data can be found on Dryad: https://doi.org/10.5061/dryad.prr4xgxws.
