# Supplementary material for: How Population Structure and Nest Membership Shape Pathogen Patterns in Bumble Bees
Source: Mol Ecol. 2025 Oct 14;34(22):e70146. doi: 10.1111/mec.70146 (PMC12617071; doi:10.1111/mec.70146)
Supplement: Supplementary file 1 — Appendix S1: PCR to distinguish between Bombus terrestris and B. lucorum. Appendix S2: Microsatellite PCRs. Table S1: Locations and sampling time points for Bombus pascuorum and B. terrestris collections in 2021 and 2022. Table S2: Primers used for microsatellite amplification. Table S3: Summary of genotyped workers, sibship reconstructions, colony density and genetic diversity in Bombus pascuorum. Table S4: Summary of genotyped workers, sibship reconstructions, colony density and genetic diversity in Bombus terrestris. Table S5: Characteristics of 12 microsatellite loci in Bombus pascuorum. Table S6: Characteristics of 12 microsatellite loci in Bombus terrestris. Table S7: PCR conditions used to screen for pathogens. Table S8: Primer sequences used for pathogen screening. Table S9: Pairwise FST between populations from island and mainland sites in Bombus pascuorum populations and B. terrestris. Table S10: Summary statistics from STRUCTURE runs using 10 subsets of the data with 119 individuals genotyped at 9 loci in Bombus pascuorum. Table S11: Summary statistics from STRUCTURE runs using 10 subsets of the data with 162 individuals genotyped at 10 loci in Bombus terrestris. Table S12: Best generalised linear mixed models or generalised linear models explaining pathogen prevalence. Table S13: Cumulative link mixed models to test whether nest membership affects pathogen dissimilarity. Table S14: Generalised linear mixed models to test whether nest membership affects individual pathogen presence in Bombus pascuorum. Table S15: Generalised linear mixed models to test whether nest membership affects individual pathogen presence in Bombus terrestris. Figure S1: Maps showing sampling sites. Figure S2: Genetic distance by geographic distance between the populations of Bombus pascuorum and B. terrestris. Figure S3: Scatterplots from ten random subsampled data sets showing the discriminant analysis of principal components (DAPC) of the first two principal components discrimin [file MEC-34-e70146-s001.pdf]

MOLECULAR ECOLOGY

Supplemental Information for:

How Population Structure and Nest Membership Shape Pathogen Patterns in Bumble Bees

Jana Dobelmann & Lena Wilfert

Institute of Evolutionary Ecology and Conservation Genomics, University of Ulm, Albert-Einstein-Allee 11, 89081 Ulm, Germany

Table of Contents:

Appendix S1: PCR to distinguish between *Bombus terrestris* and *B. lucorum*………………..…………………2
Appendix S2: Microsatellite PCRs……………………………………………………………………...2

Table S1: Locations and sampling time points for *Bombus pascuorum* and *B. terrestris* collections in 2021 and 2022.. 2
Table S2: Primers used for microsatellite amplification. 3
Table S3: Summary of genotyped workers, sibship reconstructions, colony density and genetic diversity in *Bombus pascuorum* 4
Table S4: Summary of genotyped workers, sibship reconstructions, colony density and genetic diversity in *Bombus terrestris*. 5
Table S5: Characteristics of 12 microsatellite loci in *Bombus pascuorum*. 6
Table S6: Characteristics of 12 microsatellite loci in *Bombus terrestris*. 6
Table S7: PCR conditions used to screen for pathogens. 6
Table S8: Primer sequences used for pathogen screening. 7
Table S9: Pairwise F_{ST} between populations from island and mainland sites in *Bombus pascuorum* populations and *B. terrestris* 7
Table S10: Summary statistics from STRUCTURE runs using 10 subsets of the data with 119 individuals genotyped at 9 loci in *Bombus pascuorum*.. 8
Table S11: Summary statistics from STRUCTURE runs using 10 subsets of the data with 162 individuals genotyped at 10 loci in *Bombus terrestris*.. 9
Table S12: Best generalised linear mixed models or generalised linear models explaining pathogen prevalence 10
Table S13: Cumulative link mixed models to test whether nest membership affects pathogen dissimilarity.. 11
Table S14: Generalised linear mixed models to test whether nest membership affects individual pathogen presence in *Bombus pascuorum*. 11
Table S15: Generalised linear mixed models to test whether nest membership affects individual pathogen presence in *Bombus terrestris*. 11

Figure S1: Maps showing sampling sites. 12
Figure S2: Genetic distance by geographic distance between the populations of *Bombus pascuorum* and *B. terrestris* 12
Figure S3: Scatterplots from ten random subsampled data sets showing the discriminant analysis of principal components (DAPC) of the first two principal components discriminating *Bombus pascuorum* populations. 13
Figure S4: Proportional membership of *Bombus pascuorum* island and mainland samples from 2021 and 2022 from K = 2 to K = 12 genetic clusters. 14
Figure S5: Scatterplots from ten random subsampled data sets showing the discriminant analysis of principal components (DAPC) of the first two principal components discriminating *Bombus terrestris* populations. 15
Figure S6: Proportional membership of Bombus terrestris island and mainland samples from 2021 and 2022 from K = 2 to K = 14 genetic clusters. 16
Figure S7: Scatterplot showing the correlation between *(a)* island distance from the mainland and expected heterozygosity (H_e), *(b)* island distance from the mainland and colony density or *(c)* latitude and expected heterozygosity (H_e) in *Bombus pascuorum* and *B. terrestris*. 17
Figure S8: Scatterplot showing a strong correlation between *(a)* allelic richness (AR) and expected heterozygosity (H_e) and between *(b)* colony density and H_e in *Bombus pascuorum* and *B. terrestris*. 18

Methods:

S1: PCR to distinguish between *Bombus terrestris* and *B. lucorum*

DNA was extracted from one hind leg using Chelex (5% wt/vol) and 20 µl proteinase K (100µM). PCR reactions included 1 µl 1:5 diluted DNA with 2.5 mM MgCl2 and 50 µM of each primer (BBM1_IGSF-1: GGAGCAATAATTTCAATAAATAG and BBM1_IGS_R: AARTTCAAAGCACTAATCTGC) each in a 15 µl volume. Cycling was 5 min at 94°C, followed by 35 cycles of 15 sec at 94°C, 20 s at 55°C and 20 s at 72°C, followed by 5 min at 72°C. Amplification results in a 350 bp fragment for *B. terrestris* or 286 bp for *B. lucorum*, which was visualised on a 1.5% agarose gel with RedSafe^{TM} gel stain (Intron Biotechnology).

S2: Microsatellite PCRs

Bumble bees were genotyped at 12 loci with PCRs run in two 6-plex reactions (Table S1). PCRs were run in 8-µL volumes using QIAGEN Multiplex PCR kits. PCR reactions contained 1µl 1:30 diluted DNA, 0.2 µM of each primer, except 0.3 µM for B118 and B10, and 0.4 µM for BL11 and BL132. Cycling was 15 min at 94°C, 26 cycles (28 cycles for set B in *B. terrestris*) of 30 s at 94°C, 90 s at 57°C (54°C for set A in *B. pascuorum*), and 60 s at 72°C with a final 30 min at 72°C.

Tables:

Table S1: Locations and sampling time points for *Bombus pascuorum* and *B. terrestris* collections in 2021 and 2022. * *B. terrestris* only.

Population	Site	Latitude	Longitude	Distance mainland	Collection 2021	Collection 2022
Alderney	whole island	49.71321	-2.20692	15 km	22. – 24. August	22. – 24. June and 5. - 6. August
Arran	Brodick	55.59502	-5.15081	5 km	26. August*	1. August – 3. August
	Lagg	55.44560	-5.23609		-	31. July -2. August
	Lamlash	55.53835	-5.12319		-	29. July
	Lochranza	55.70363	-5.29064		-	30. July – 2. August
	Whiting	55.48660	-5.09762		-	29. - 2. August
Belle-Ile	Bangor	47.31594	-3.1895	13 km	13. – 15. June	10. – 11. July
	Borthelo	47.33488	-3.15398		15. – 17. June	-
	Calastren	47.30636	-3.17372		15. – 17. June	-
	Grand Cosquet	47.30025	-3.1376		-	10. - 15. July
	Le Palais	47.34712	-3.15538		15. – 17. June	11. – 14. July
	Locmaria	47.29500	-3.08533		-	13. – 14. July
	Petit Cosquet	47.31490	-3.21221		15. – 17. June	-
	Sauzon	47.37061	-3.22236		-	12. – 14. July
Guernsey	Icart	49.42188	-2.56091	45 km	-	14. – 24. June
	Peter Port	49.45977	-2.53468		21. – 25. August	17. June
	Pierre du Bois	49.44173	-2.64121		-	18. June
	Saumarez	49.47105	-2.57881		-	14. – 16. June
	Vale	49.49811	-2.52697		-	16. – 24. June
Isle of Man	Castletown	54.07490	-4.65438	30 km	-	8. August
	Dalby	54.15540	-4.70825		-	9. August
	Douglas	54.15243	-4.48584		19. - 23. July	10. August
	Ramsey	54.32432	-4.39203		-	6. – 7. August
	St Johns	54.20323	-4.64283		-	6. August
Ouessant		48.45746	-5.09644	20 km	22. -26. June	4. – 7. July
Scilly Isles*		49.91428	-6.30417	45 km	20. July	-
Le Conquet		48.35945	-4.77332	-	22. and 26. June	-
Liverpool		53.40759	-2.99091	-	19. – 23. July	-
Penryn		50.15276	-5.06717	-	19. – 23. July	-
Quiberon*		47.4822	-3.12019	-	21. June	-

Table S2: Primers used for microsatellite amplification. Bp shows primers that were used for amplification in *Bombus pascuorum* and Bt shows those used for amplification of *B. terrestris* and the multiplex reaction the primers were used in (A or B). The forward primer was labelled with a fluorescent dye.

Primer	Dye	Bp	Bt	Concs. (μM)	Sequence (5' – 3')	Repeat structure	Reference
B10	6-Fam	B	B	0.25	F: GTGTAACTTTCTCTCGACAG<br>R: GGGAGATGGATATAGATGAG	$(CT)_4TT(CT)_{13}$	Estoup et al. (1996); Estoup et al. (1995)
B11	Ned	B	B	0.17	F: GCAACGAAACTCGAAATCG<br>R: GTTCATCCAAGTTTCATCCG	$(CT)_5…(CT)_{10}(ATCT)_6…(CT)_3$	
B96	Vic	A	A	0.17	F: GGGAGAGAAAGACCAAG<br>R: GATCGTAATGACTCGATATG	$(CG)_3(CT)_6TT(CT)_3T(CT)_4T(CT)_7$	
B100	Vic		B	0.17	F: CGTCCTCGTATCGGGCTAAC<br>R: CGTGGAAACGTCGTGACG	$(CT)_{12}GTC(CT)_3$	
B118	6-Fam	A	A	0.25	F: CCTAAGTCGCTATATCTTCG<br>R: GAAACACGTATCTACATCTACAG	$(CT)_{11}AG(CT)_3$	
B119	6-Fam		B	0.17	F: GATCGTGCTAGAAAAGGAAG<br>R: CCACAGTGCAAAGTTTCTG	$(CT)_7CG(CT)_4$	
B121	Vic	B		0.17	F: GAACATGTGGAACGACGG<br>R: GAACAATCGATATGTCACCG	$(CT)_2TT(CT)_3TTCTTTCC(CT)_4CCTT(CT)_4CC(CT)_6$	
B124	Vic	B	B	0.17	F: GCAACAGGTCGGGTTAGAG<br>R: CAGGATAGGGTAGGTAAGCAG	$(CT)_8TCCTCTTCCAC(CT)_{14}CCTC(GC)_?...(GGCT)_8$	
B126	Ned	A	A	0.17	F: GCTTGCTGGTGAATTGTGC<br>R: CGATTCTCTCGTGTACTCC	$(CT)_{12}GT(CT)_{10}$	
B131	6-Fam	B		0.17	F: GATCGCCTATCTCTTCTCGG<br>R: GAGGCGCTGTCGAGCTC	$(CT)_4TT(TC)_4$	
B132	Pet	B	B	0.33	F: GAAATTCGTGCGGAGGG<br>R: CAGAGAACTACCTAGTGCTACGC	$(CT)_{12}TC(CT)_3$	
BL03	Vic	A	A	0.17	F: CGAAAATCAGGGGTGACAAAC<br>R: CCTTTCTGTTTATAGTTCGTCCG	$(AG)_{25}$	Reber Funk et al. (2006)
BL06	6-Fam	A	A	0.17	F: CCCGCTCGATATTACGATG<br>R: CCCCTATCCCATTGATACGC	$(CT)_{24}$	
BL11	Pet	A	A	0.33	F: AAGGGTACGAAATGCGCGAG<br>R: TGACGAGTGCGGCCTTTTTC	$(TG)_{21}$	

Table S3: Summary of genotyped workers, sibship reconstructions, colony density and genetic diversity in *Bombus pascuorum*. W1 to w6 show how many nests with 1 to 6 sisters were detected. Population sizes were calculated using mark-recapture models for 2022 samples using the equal capture model (ECM) or †the two innate rates model (TIRM). AR: rarefied allelic richness (sites with fewer than 10 colonies excluded), H_e: expected heterozygosity, F_{IS}: inbreeding coefficient.

Population	Year	Site	Workers	Colonies	w1	w2	w3	w4	w5	w6	Pop. size (95% CI)	Nests km^{-2}	Mean nests km^{-2}	AR	H_e	Mean H_e pop.	Mean F_{IS} pop.
Alderney	2021	Whole island	32	30	28	2	0	0	0	0	NA	NA	NA	3.9	0.495		
	2022	August	73	59	45	14	0	0	0	0	163 (106 – 267)	21	22	4.2	0.517	0.507	0.042
		June	49‡	43	38	4	1	0	0	0	179 (90 – 479)	23		4.1	0.510		
Arran	2021	Brodick	36	34	32	2	0	0	0	0	NA	NA	NA	5.7	0.601		
	2022	Brodick	42	35	29	5	1	0	0	0	109 (72 – 273)	29		5.9	0.604		
		Lagg	41	28	18	8	1	1	0	0	49 (33 – 77)	13		5.1	0.575		
		Lamlash	42	36	30	6	0	0	0	0	129 (87 – 273)	34	29	6.0	0.609	0.599	0.072
		Lochranza	33	29	26	2	1	0	0	0	121 (64 – 517)	32		5.2	0.611		
		Whiting	58	48	38	10	0	0	0	0	146 94 – 285)	38		5.6	0.594		
Belle-Ile	2021	Whole island	4	4	4	0	0	0	0	0	NA	NA	NA	NA	0.698		
	2022	Bangor	40	31	26	3	0	2	0	0	108† (68 – 198)	28		5.0	0.642		
		Gr. Cosquet	32	23	19	1	2	0	1	0	70† (44 – 168)	18		5.4	0.657		
		Le Palais	39	22	11	8	2	0	0	1	32 (23 – 47)	8	20	5.4	0.667	0.667	0.035
		Locmaria	44	37	31	5	1	0	0	0	120 (79 – 263)	32		5.5	0.674		
		Sauzon	31	24	18	5	1	0	0	0	56 (36 – 145)	15		5.2	0.666		
Guernsey	2021	Peter Port	29	24	21	2	0	1	0	0	NA	NA	NA	3.7	0.372		
	2022	Icart	39 (7‡)	31	24	6	1	0	0	0	79 (43 – 172)	21		4.3	0.444		
		Peter Port	42	30	24	2	3	0	1	0	88† (65 – 218)	23		4.2	0.386		
		Pierre du Bois	38 (19‡)	32	26	6	0	0	0	0	104 (57 – 222)	27	25	3.9	0.400	0.407	0.020
		Saumarez	42	32	26	4	1	0	1	0	103† (70 – 235)	27		4.4	0.442		
		Vale	35 (21‡)	27	23	2	1	0	1	0	96† (64 – 303)	25		3.9	0.397		
Isle of Man	2021	Douglas	35	21	15	2	2	1	0	1	NA	NA	NA	4.0	0.491	0.515	0.022
	2022	Castletown	40	26	16	7	2	1	0	0	42 (29 – 61)	11		3.8	0.515		
		Dalby	42	36	33	1	1	1	0	0	192† (105 – 870)	50		4.0	0.499		
		Douglas	43	35	28	6	1	0	0	0	98 (51 – 211)	26	25	4.4	0.563		
		Ramsey	39	29	21	6	2	0	0	0	61 (39 – 93)	16		4.1	0.513		
		St Johns	44	31	23	4	3	1	0	0	82† (61 – 165)	22		4.0	0.508		
Ouessant	2021	Whole island	35	25	17	6	2	0	0	0	NA	NA	NA	6.5	0.740	0.727	0.198
	2022	Whole island	57	41	28	11	1	1	0	0	80 (54 – 126)	5	5	6.1	0.714		
Le Conquet	2021		25	17	13	2	1	0	1	0	NA	NA	NA	7.9	0.707		0.034
Liverpool	2021		11	6	4	0	1	1	0	0	NA	NA	NA	NA	0.729		NA
Penryn	2021	Campus	9	8	7	1	0	0	0	0	NA	NA	NA	NA	0.632		NA
		Total	1201	934	774	150	34	10	6	2	Mean		23.64	4.9	0.568		

‡Defrosted samples not used in pathogen screening.

Table S4: Summary of genotyped workers, sibship reconstructions, colony density and genetic diversity in *Bombus terrestris*. W1 to w10 show how many nests with 1 to 10 sisters were detected. Population sizes were calculated using mark-recapture models for 2022 samples using the equal capture model (ECM) or † the two innate rates model (TIRM). AR: rarefied allelic richness (sites with fewer than 10 colonies excluded), H_e: expected heterozygosity, F_{IS}: inbreeding coefficient.

Population	Year	Site	Workers	Colonies	w1	w2	w3	w4	w5	w6	w10	Pop. size (95% CI)	Nests km^{-2}	Mean nests km^{-2}	AR	H_e	Mean H_e pop.	Mean F_{IS} pop.
Alderney	2021	Whole island	35	29	25	3	0	1	0	0	0	NA	NA		7.3	0.794	0.786	0.009
	2022	August	59	50	41	9	0	0	0	0	0	170 (102 – 367)	22	34	7.0	0.787		
		June	62‡	57	53	3	1	0	0	0	0	357 (189 – 925)	46		7.2	0.778		
Arran	2021	Brodick	9	9	9	0	0	0	0	0	0			NA	NA	0.749	0.784	-0.019
	2022	Brodick	18	13	9	3	1	0	0	0	0	24 (13 – 71)	3	9	7.9	0.807		
		Lagg§	9	8	7	1	0	0	0	0	0	33 (9 – 1000)	NA		NA	0.767		
		Lamlash§	20	20	20	0	0	0	0	0	0	NA	NA		8.1	0.799		
		Lochranza§	1	1	1	0	0	0	0	0	0	NA	NA		NA	NA		
		Whiting	22	20	18	2	0	0	0	0	0	108 (50 – 1000)	15		8.2	0.798		
Belle-Ile	2021	Bangor	49	40	32	7	1	0	0	0	0	NA	NA	NA	7.7	0.800	0.794	0.036
		Borthelo	57	50	45	3	2	0	0	0	0				7.5	0.789		
		Calastren	44	40	36	4	0	0	0	0	0				7.1	0.794		
		Le Palais	48	44	40	4	0	0	0	0	0				7.5	0.799		
		Pt. Cosquet	52	41	33	5	3	0	0	0	0				7.5	0.791		
	2022	Bangor	46	34	26	4	4	0	0	0	0	70 (48 – 107)	9	16	7.5	0.798		
		Gr. Cosquet	42	30	24	3	2	0	0	1	0	78 (51 – 145)	10		7.3	0.783		
		Le Palais	45	36	30	4	1	1	0	0	0	129† (86 – 296)	17		7.6	0.805		
		Locmaria	44	36	29	6	1	0	0	0	0	103 (64 – 263)	14		7.4	0.785		
		Sauzon	37	33	31	1	0	1	0	0	0	225† (121 – 1000)	30		7.4	0.799		
Guernsey	2021	Peter Port	14	13	12	1	0	0	0	0	0	NA	NA	NA	7.8	0.811	0.814	0.013
	2022	Icart	39	35	31	4	0	0	0	0	0	172 (79 – 728)	23	38	8.0	0.819		
		Peter Port	39	37	35	2	0	0	0	0	0	358 (172 – 1000)	48		7.7	0.821		
		Pierre du Bois§	36 (27‡)	36	36	0	0	0	0	0	0	NA	NA		7.6	0.808		
		Saumarez	43	41	39	2	0	0	0	0	0	437 (166 – 1000)	59		7.6	0.809		
		Vale	37	33	29	4	0	0	0	0	0	154 (77 – 654)	21		8.0	0.817		
Isle of Man	2021	Douglas	18	17	16	1	0	0	0	0	0	NA	NA	NA	5.6	0.645	0.672	0.081
	2022	Castletown	44	34	28	3	2	1	0	0	0	113† (773 – 258)	15	16	5.2	0.663		
		Dalby§	42	41	40	1	0	0	0	0	0	847 (273 – 1000)	NA		5.3	0.667		
		Douglas	46	36	30	4	1	0	1	0	0	125† (92 – 248)	17		5.8	0.701		
		Ramsey	50	43	37	5	1	0	0	0	0	158 (94 – 392)	21		5.5	0.673		
		St Johns	36	28	21	6	1	0	0	0	0	66 (42 – 144)	9		5.4	0.682		
Ouessant	2021	Whole island	60	22	7	8	1	3	0	2	1	NA	NA	NA	7.7	0.802	0.804	0.035
	2022	Whole island	75	53	39	11	0	1	2	0	0	139† (114 – 234)	9	9	7.6	0.806		
Scilly Isles	2021	St Mary's	35	34	33	1	0	0	0	0	0	NA	NA	NA	4.9	0.646		0.039
Le Conquet	2021		53	38	27	7	4	0	0	0	0	NA	NA	NA	8.5	0.818		0.055
Liverpool	2021		12	12	12	0	0	0	0	0	0	NA	NA	NA	7.1	0.791		0.064
Penryn	2021	Campus	21	18	16	1	1	0	0	0	0	NA	NA	NA	7.2	0.776		0.075
Quiberon	2021		38	24	15	5	3	1	0	0	0	NA	NA	NA	8.0	0.810		-0.003
		Total	1437	1181	1002	133	30	9	3	1	1	Mean		21.55	7.2	0.766		

‡Defrosted samples not used in pathogen screening. §Too few sisters were found and excluded from pathogen dissimilarity analysis and population size estimation.

Table S5: Characteristics of 12 microsatellite loci in *Bombus pascuorum*. The range of amplicon length, null allele frequency, observed heterozygosity (H_o) and expected heterozygosity (H_e), number of alleles (A), inbreeding coefficient (FIS), failed amplifications and error rate are shown for each locus. Error rates were calculated from three individuals that were included in every PCR and sequencing run. B118 and was excluded from the analysis in both years, additionally, B124 in 2021 and B10 in 2022 as it failed amplification in 57 samples from Ouessant.

Locus	Range	Null allele	H_o	H_e	A	FIS	Failed amplification	Error rate
BL06	131 – 155	0.065	0.29	0.38	10	0.154	0.1%	0.057
BL03	127 – 173	0.076	0.7	0.84	21	0.038	0.1%	0.029
B96	210 – 256	0.071	0.57	0.69	15	0.053	0.1%	0.056
B126	123 – 145	0.109	0.31	0.47	8	0.094	0.2%	0.000
BL11	117 – 139	0.080	0.49	0.62	11	0.063	1.3%	0.000
B131	119 – 149	0.061	0.7	0.81	15	0.029	0.2%	0.000
B10*	170 - 201	0.197	0.1	0.37	9	0.658	4.8%	0.000
B118	214 - 240		0.458	-	14	-	0.1%	0.000
B121	129 - 179	0.089	0.74	0.91	24	0.030	0.2%	0.063
B124*	236 – 268	0.200	0.4	0.75	13	0.099	0.7%	0.000
B11	125 - 161	0.024	0.24	0.27	10	0.043	0.2%	0.000
B132	145 – 175	0.095	0.62	0.79	12	0.094	1.7%	0.029

Table S6: Characteristics of 12 microsatellite loci in *Bombus terrestris*. The range of amplicon length, null allele frequency, observed heterozygosity (H_o) and expected heterozygosity (H_e), number of alleles (A), inbreeding coefficient (FIS), failed amplifications and error rate are shown for each locus. Error rates were calculated from three individuals that were included in every PCR and sequencing run. B119 was excluded from the analysis when using 2021 data as we only found 4 alleles that year and 57% homozygotes.

Locus	Range	Null allele	H_0	H_e	A	FIS	Failed amplification	Error rate
BL06	143 – 193	0.027	0.83	0.88	23	0.005	0%	0.000
B118	206 – 224	0.017	0.72	0.75	10	0.036	0.3%	0.000
BL03	129 – 169	0.027	0.83	0.88	20	0.003	0%	0.000
B96	225 – 269	0.050	0.53	0.61	14	0.029	0.1%	0.033
B126	137 – 209	0.059	0.74	0.85	29	0.106	0.1%	0.000
BL11	133 – 201	0.058	0.79	0.9	28	0.028	0.8%	0.000
B119*	119 – 135	0.232	0.48	0.68	8	0.092	0.1%	0.030
B10	178 – 252	0.037	0.83	0.9	28	0.014	0.6%	0.121
B100	141 – 197	0.039	0.72	0.79	19	0.042	0%	0.029
B124	226 – 282	0.037	0.82	0.89	22	0.054	0.4%	0.118
B11	139 - 117	0.029	0.69	0.74	17	-0.026	0.6%	0.000
B132	153 - 189	0.033	0.78	0.84	17	0.048	0.5%	0.000

Table S7: PCR conditions used to screen for black queen cells virus (BQCV), deformed wing virus type A and B (DWV-A and DWV-B), slow bee paralysis virus (SBPV), *Apicystis bombi*, *Crithidia bombi* and *Nosema* spp.

Pathogen	React. volume [µl]	cDNA (1:30) [µl]	Polymerase [U]	dNTPs [mM]	Primer [µM]	$MgCl_2$ [mM]	Amplification programme
BQCV	15	3	0.5	0.2	0.5	2.5	94°C 2min, 40x 15s 94°C, 30s 56°C, 30s 72°C, final 7 min 72°C
DWV-A	15	3	0.375	0.2	0.5	2.5	94°C 2min, 38x 20s 94°C, 30s 55°C, 45s 72°C, final 5 min 72°C
DWV-B	15	3	0.375	0.2	0.5	2.5	94°C 2min, 35x 20s 94°C, 30s 60°C, 30s 72°C, final 5 min 72°C
SBPV	15	3	0.75	0.2	0.5	2.5	94°C 2min, 37x 15s 94°C, 20s 58°C, 30s 72°C, final 7 min 72°C
A. bombi	15	3	1.25	0.4	0.5	1.5	94°C 2min, 35x 30s 94°C, 30s 60.7°C, 45s 72°C, final 3 min 72°C
C. bombi	15	3	0.5	0.125	0.2	2.5	95°C 5 min, 40x 95°C 30s, 55°C 30s, 72°C1 min, final 72°C 10 min
Nosema spp.	20	3	0.5	0.3	0.4 (0.5 (*N. bombi* F & *Nosema* univ. R)	3	95°C 2min, 40x 95°C 30s, 55°C 30s, 72°C 1min, final 72°C 5min

Table S8: Primer sequences used for pathogen screening.

Pathogen	Primer	Sequence (5’ – 3’)	Amplicon length (bp)	Reference
BQCV	F	TCCYCCAGTTCAACCATCTA	1257	Manley et al. (2020)
	R	AACGTTGCCTAGRTTCGTCA		
DWV-A	F	AAATCCGGTATGGGAAGTCATGC	929	Dobelmann et al. (2024)
	R	CTGCCTGAGCTTCTCTAACTTCG		
DWV-B	F	GAGATATGGAAGTTCGAGTGCAGA	405	
	R	TTGCCCTAATTTGATTCGCGTAAA		
SBPV	F	GCAGCCTGAAATGATGATGTC	424	Lena Wilfert (*pers. comm.*)
	R	GTTCGGTCGGTTGGTGAAAG		
A. bombi	F	CCAGCATGGAATAACATGTAAGG	679	Meeus et al. (2014)
	R	GACAGCTTCCAATCTCTAGTCG		
C. bombi	F	GGAAACCACGGAATCACATAGACC	260	Schmid-Hempel and Tognazzo (2010)
	R	AGGAAGCCAAGTCATCCATCGC		
Nosema apis	F	GCATGTCTTTGACGTACTATG	224	Fries et al. (2013)
N. bombi	F	TTTATTTTATGTRYACMGCAG	171	
N. ceranae	F	CGTTAAAGTGTAGATAAGATGTT	143	
Nosema spp.	R	GACTTAGGAGTAGCCGTCTCTC		

Table S9: Pairwise F_{ST} between populations from island and mainland sites in *Bombus pascuorum* (upper triangle) and *B. terrestris* (lower triangle) populations. Mainland sites are in blue and sampled in 2021, islands were sampled in 2021 and 2022, except for the Scilly Isles, which were only sampled in 2021. No *B. pascuorum* were sampled in Quiberon or on the Scilly Isles. Moderate differentiation (F_{ST}: 0.05 – 0.15) in light grey, high (0.15 - 0.25) in grey and very high (> 0.25) in dark grey and white font.

F_{ST}	Alderney	Arran	Belle-Ile	Guernsey	Isle of Man	Ouessant	Le Conquet	Liverpool	Penryn	Quiberon
Alderney	-	0.087	0.180	0.131	0.152	0.208	0.077	0.097	0.087	-
Arran	0.046	-	0.110	0.171	0.074	0.143	0.031	0.013	0.016	-
Belle-Ile	0.043	0.052	-	0.254	0.184	0.155	0.070	0.097	0.103	-
Guernsey	0.015	0.045	0.023	-	0.234	0.285	0.157	0.187	0.164	-
Isle of Man	0.122	0.067	0.109	0.111	-	0.181	0.095	0.080	0.093	-
Ouessant	0.034	0.048	0.024	0.023	0.097	-	0.103	0.065	0.121	
Le Conquet	0.035	0.047	0.014	0.020	0.093	0.011	-	0.015	0.013	-
Liverpool	0.052	0.026	0.054	0.043	0.104	0.061	0.045	-	0.000	-
Penryn	0.036	0.024	0.045	0.033	0.113	0.051	0.044	0.005	-	-
Quiberon	0.040	0.043	0.018	0.018	0.109	0.020	0.016	0.034	0.027	-
Scilly-Isles	0.153	0.114	0.135	0.13	0.190	0.140	0.141	0.116	0.093	0.124

Table S10: Summary statistics from STRUCTURE runs using 10 subsets of the data with 119 individuals (max. 15 per population) genotyped at 9 loci in *Bombus pascuorum*. Optimal K was estimated using the log probability of the data lnP(D) and the Evanno Method (ΔK), highest lnP(D) is marked in dark grey and highest ΔK in light grey, the optimal K for each subset using both methods is stated in the summary column (*), whereby 'ghost clusters' with a mean membership coefficient < 0.5 in any population have been removed (Puechmaille, 2016) and the corrected ΔK and PP are reported. Clusters that form at this optimal K are separated by "|" with letters indicating populations within clusters: A: Alderney, B: Belle-Ile, C: Le Conquet, G: Guernsey, L: Liverpool, M: Isle of Man, O: Ouessant, P: Penryn, R: Arran.

K	runs	Method	sub1	sub2	sub3	sub4	sub5	sub6	sub7	sub8	sub9	sub10
1	10	lnP(D)	-3625	-3483	-3650	-3640	-3592	-3478	-3643	-3516	-3515	-3611
2	10	ΔK	**243.36**	10.28	**251.02**	**198.04**	1.60	3.17	**316.95**	7.47	0.28	0.01
		lnP(D)	-3284	-3311	-3266	-3226	-3735	-3333	-3244	-3348	-3330	-3381
3	10	ΔK	1.40	**176.87**	1.32	45.67	**180.78**	**241.84**	1.69	1.10	0.92	3.84
		lnP(D)	-3200	-3039	-3161	-3069	-3159	-3051	-3107	-3150	-3157	-3151
4	10	ΔK	1.72	31.14	4.22	11.87	0.59	25.92	0.86	**39.79**	**14.30**	8.05
		lnP(D)	-3091	-2968	-3077	-2978	-3159	-2998	-3071	-3045	-3047	-3065
5	10	ΔK	1.52	0.81	4.66	3.65	1.38	4.95	1.02	0.95	1.23	4.37
		lnP(D)	-3002	-3067	-3016	-2928	-3198	-3078	-3022	-3069	-2999	-3070
6	10	ΔK	23.27	0.18	0.22	3.07	0.45	30.46	30.50	0.88	0.29	**36.55**
		lnP(D)	**-2958**	-2984	-2996	-2909	-3141	**-2982**	**-2941**	-2985	-2986	**-2957**
7	10	ΔK	0.45	4.45	0.87	0.29	0.85	1.17	2.28	0.84	1.11	1.41
		lnP(D)	-3045	**-2880**	**-2971**	**-2908**	**-3037**	-3131	-3003	**-2949**	**-2952**	-3041
8	10	ΔK	0.84	1.14	0.09	1.78	2.78	0.14	0.35	0.38	0.47	0.23
		lnP(D)	-3111	-2921	-3002	-2924	-3061	-3092	-3122	-3008	-3019	-3038
9	10	ΔK	3.08	0.07	0.68	0.29	1.78	7.00	0.22	1.42	0.01	1.00
		lnP(D)	-3050	-2991	-3046	-3011	-3186	-3070	-3189	-3051	-3062	-3055
10	10	ΔK	0.15	1.48	3.34	0.09	3.06	1.48	1.37	0.95	1.37	0.15
		lnP(D)	-3152	-3059	-3037	-3079	-3222	-3214	-3284	-3142	-3105	-3129
11	10	ΔK	1.29	0.96	0.33	0.34	1.39	1.19	7.94	0.05	0.96	0.37
		lnP(D)	-3265	-3190	-3111	-3143	-3356	-3194	-3164	-3175	-3213	-3192
12	10	lnP(D)	-3268	-3225	-3142	-3179	-3377	-3266	-3255	-3210	-3239	-3226
Optimal		ΔK	2	3	2	2	3	2 (3*)	2	3 (4*)	4	5 (6*)
		Clusters	O | ABCGL MPR	O | AG | BCLMPR	O | ABCGL MPR	O | ABCGL MPR	O | AG | BCLMP R	AG | BCLM OPR	O | ABCGL MPR	O | AG | M | BCLPR	O | AG | M | BCLPR	O | AG | M | B | CLPR
Optimal		lnP(D)	5 (6*)	4 (7*)	5 (7*)	5 (7*)	5 (7*)	3 (6*)	5 (6*)	4 (7*)	4 (7*)	5 (6*)
		Clusters	O | AG | M | B | CLPR	O | AG | B | CLMPR	O | AG | M | B | CLPR	O | AG | M | B | CLPR	O | AG | M | B | CLPR	O | AG | BCLMP R	O | AG | M | B | CLPR	O | AG | M | BCLPR	O | AG | M | BCLPR	O | AG | M | B | CLPR

Table S11: Summary statistics from STRUCTURE runs using 10 subsets of the data with 162 individuals (max. 15 per population) genotyped at 10 loci in *Bombus terrestris*. Optimal K was estimated using the log probability of the data lnP(D) and the Evanno Method (ΔK), highest lnP(D) is marked in dark grey and highest ΔK in light grey, the optimal K for each subset using both methods is stated in the summary column (*), whereby 'ghost clusters' with a mean membership coefficient < 0.5 in any population have been removed (Puechmaille, 2016) and the corrected ΔK and PP are reported. Clusters that form at this optimal K are separated by "|" with populations grouped into regions: UK: Arran, Liverpool, Penryn, Isle of Man (M), Scilly Isles (S) and France: Belle-Ile, Quiberon, Ouessant, Le Conquet, including the Channel Islands Alderney and Guernsey.

K	runs	Method	sub1	sub2	sub3	sub4	sub5	sub6	sub7	sub8	sub9	sub10
1	10	lnP(D)	-7480	-7419	-7432	-7544	-7480	-7535	-7442	-7411	-7543	-7560
2	10	ΔK	**83.20**	**180.89**	**134.59**	**94.84**	15.60	**180.79**	**80.52**	**132.81**	**16.63**	**45.12**
		lnP(D)	-7265	-7163	-7205	-7282	-7262	-7329	-7270	-7192	-7352	-7347
3	10	ΔK	0.50	9.29	0.38	55.22	**132.40**	0.83	0.80	0.72	8.71	0.77
		lnP(D)	-7249	-7225	-7216	**-7131**	**-7084**	-7569	-7419	-7293	-7212	-7303
4	10	ΔK	10.90	0.45	8.87	3.55	1.23	34.29	11.03	6.23	9.52	1.60
		lnP(D)	-7097	-7136	**-7079**	-7165	-7255	**-7183**	-7176	**-7141**	-7212	**-7224**
5	10	ΔK	17.10	0.28	0.42	0.19	0.81	0.65	1.35	0.76	1.20	0.33
		lnP(D)	**-7018**	-7130	-7253	-7245	-7579	-7332	**-7169**	-7279	-7329	-7275
6	10	ΔK	7.22	1.90	0.34	0.54	4.72	1.45	1.56	1.71	4.94	0.18
		lnP(D)	-7078	**-7106**	-7481	-7197	-7286	-7321	-7216	-7219	**-7187**	-7376
7	10	ΔK	1.31	0.90	0.28	0.58	0.05	0.10	1.04	0.01	2.18	0.22
		lnP(D)	-7361	-7257	-7583	-7308	-7373	-7544	-7411	-7399	-7557	-7419
8	10	ΔK	0.94	0.94	0.11	1.37	0.22	0.88	0.17	0.26	0.67	2.81
		lnP(D)	-7356	-7554	-7569	-7338	-7463	-7783	-7804	-7577	-8432	-7420
9	10	ΔK	0.60	0.51	0.16	1.23	1.07	1.50	0.87	0.24	7.60	1.05
		lnP(D)	-7623	-7695	-7512	-7547	-7531	-7674	-8297	-7700	-7745	-7754
10	10	ΔK	0.07	0.95	0.05	0.37	1.45	0.23	1.64	0.11	1.03	0.02
		lnP(D)	-7741	-7740	-7504	-7484	-7447	-7821	-7970	-7759	-8643	-7851
11	10	ΔK	0.16	0.48	1.61	2.53	0.44	2.75	0.64	0.62	1.27	0.26
		lnP(D)	-7830	-7881	-7509	-7493	-7642	-8029	-8144	-7853	-8217	-7952
12	10	ΔK	0.78	0.04	0.62	0.80	0.24	0.71	0.45	0.88	0.23	0.53
		lnP(D)	-7870	-8133	-7760	-7895	-7722	-9076	-8136	-7648	-8276	-8159
13	10	ΔK	0.13	0.66	0.43	0.13	0.66	2.65	0.24	0.88	2.07	0.58
		lnP(D)	-8068	-8371	-7749	-7902	-7758	-8122	-8272	-7650	-8199	-8034
14	10	lnP(D)	-8315	-8270	-7839	-7852	-8017	-8425	-8541	-7791	-8680	-8109
Optimal		ΔK	2	2	2	2	3	2	2	2	2	2
		Clusters	UK | France	UK | France	UK | France	UK | France	S | UK | France	UK | France	UK | France	UK | France	UK | France	UK | France
Optimal		lnP(D)	4 (5*)	3 (6*)	4	3	3	3 (4*)	3 (5*)	4	4 (6*)	4
		Clusters	S | M | UK | France	S | UK | France	S | M | UK | France	S | UK | France	S | UK | France	S | UK | France	S | UK | France	S | M | UK | France	S | M | UK | France	S | M | UK | France

Table S12: Best generalised linear mixed models (GLMMs) or generalised linear models (GLMs) explaining *Apicystis bombi*, *Crithidia bombi*, *Nosema bombi*, deformed wing virus type B (DWV-B) and slow bee paralysis virus (SBPV) prevalence in *Bombus pascuorum* and *B. terrestris*. GLMMs were fitted with a binomial error structure and logit link function, using island as a random effect. We used the *anova()* function to test whether including this random effect improved the model fit and used generalised linear models (GLMs) when the random effect could be omitted. For *C. bombi* in *B. terrestris*, *N. bombi* in *B. pascuorum* and DWV-B in both species GLMs were used. H_e: expected heterozygosity.

		B. pascuorum				*B. terrestris*			
Pathogen	Predictor	Estimate	SE	z-value	P-value	Estimate	SE	z-value	P-value
A. bombi	Intercept	-2.373	0.696	-3.410	0.001	-1.513	0.312	-4.845	0.000
	Varroa pres.	1.868	0.848	2.202	0.028	1.159	0.371	3.126	0.002
	Colonies km^{-2}	0.355	0.098	3.611	<0.001	0.291	0.116	2.501	0.012
C. bombi	Intercept	-2.189	0.312	-7.015	<0.001	-0.170	0.088	-1.940	0.052
	Latitude	-0.797	0.331	-2.405	0.016				
	Colonies km^{-2}					0.684	0.097	7.082	0.000
	H_e	-0.545	0.236	-2.303	0.021				
	N. bombi	0.943	0.340	2.775	0.006	0.822	0.205	4.014	0.000
N. bombi	Intercept	-1.943	0.235	-8.252	<0.001	-3.138	0.763	-4.111	0.000
	Varroa pres.	-2.879	0.388	-7.421	<0.001				
	Latitude	1.034	0.261	3.959	<0.001				
	C. bombi	1.087	0.339	3.202	0.001	0.663	0.220	3.017	0.003
DWV-B	Intercept	-1.443	0.173	-8.344	<0.001	-2.939	0.403	-7.289	0.000
	Varroa pres.	1.031	0.207	4.975	<0.001	2.782	0.531	5.240	0.000
	Latitude	-0.293	0.075	-3.879	<0.001				
	Colonies km^{-2}					0.284	0.089	3.203	0.001
	H_e	0.385	0.077	4.990	<0.001	-0.704	0.240	-2.928	0.003
	A. bombi	0.331	0.164	2.020	0.043				
	SBPV					0.576	0.196	2.944	0.003
SBPV	Intercept	-0.939	0.609	-1.541	0.123	-1.231	0.643	-1.915	0.056
	Latitude	1.666	0.632	2.637	0.008	2.391	0.729	3.281	0.001
	Colonies km^{-2}	0.541	0.104	5.226	<0.001				
	H_e	1.307	0.393	3.329	0.001	2.254	0.546	4.130	0.000
	DWV-B					0.551	0.217	2.542	0.011

Table S13: Cumulative link mixed models to test whether nest membership affects pathogen dissimilarity. Models were fitted by using pairwise pathogen dissimilarities (range 0 to 5) as the ordinal response with pairing (sisters or non-sisters) as a predictor and IDs of both individuals as random effects. The random intercept variance in the *B. pascuorum* model was 2.619 (SD = 1.618) for ID1 and 2.456 (SD=1.567) for ID2. For *B. terrestris*, this random intercept variance was 2.619 (SD = 1.149) for ID1 and 1.112 (SD=1.055) for ID2.

Host species	Predictor	Estimate	SE	z-value	P-value
B. pascuorum	Pairing: Sisters	-0.225	0.131	-1.712	0.087
B. terrestris	Pairing: Sisters	-0.453	0.154	-2.938	0.003

Table S14: Generalised linear mixed models to test whether nest membership affects individual pathogen presence in *Bombus pascuorum*. Binomial models (1: different infection status or 0: same infection status) were fitted with pairing (sisters or non-sisters) as a predictor and IDs of both individuals as random effects. ±Not significant using Benjamini-Hochberg corrected significance thresholds.

Model	Predictor	Estimate	SE	z-value	P-value
A. bombi	Intercept	-2.000	0.102	-19.680	<0.001
	Sisters	0.060	0.187	0.320	0.749
C. bombi	Intercept	-2.697	0.121	-22.346	<0.001
	Sisters	-0.055	0.212	-0.249	0.803
N. bombi	Intercept	-6.162	0.251	-24.562	<0.001
	Sisters	-0.292	0.317	-0.921	0.357
DWV-B	Intercept	-1.261	0.267	-4.724	<0.001
	Sisters	-0.391	0.180	-2.170	0.030±
SBPV	Intercept	-3.586	0.150	-23.938	<0.001
	Sisters	-0.287	0.236	-1.215	0.224

Table S15: Generalised linear mixed models to test whether nest membership affects individual pathogen presence in *Bombus terrestris*. Binomial models (1: different infection status or 0: same infection status) were fitted with pairing (sisters or non-sisters) as a predictor and IDs of both individuals as random effects. Significant p-values are indicated by *.

Model	Predictor	Estimate	SE	z-value	P-value
A. bombi	Intercept	-1.254	0.088	-14.272	<0.001
	Sisters	-0.324	0.215	-1.509	0.131
C. bombi	Intercept	-0.492	0.053	-9.207	<0.001
	Sisters	-0.461	0.177	-2.609	0.009*
N. bombi	Intercept	-3.396	0.152	-22.322	<0.001
	Sisters	-1.254	0.342	-3.665	0.0002*
DWV-B	Intercept	-1.936	1.225	-1.580	0.114
	Sisters	0.198	0.186	1.064	0.287
SBPV	Intercept	-2.061	0.108	-19.095	<0.001
	Sisters	0.0004	0.222	0.002	0.998

Figures

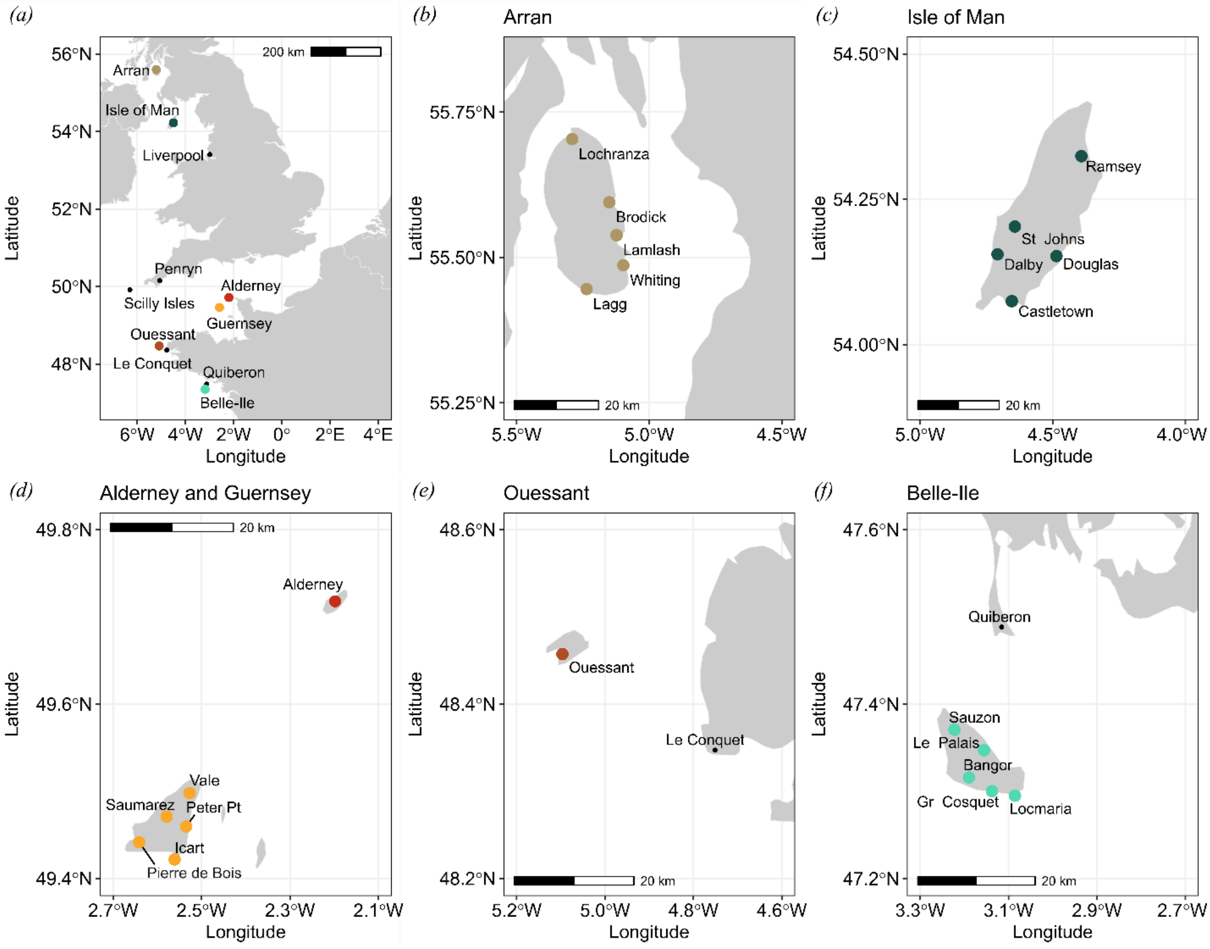

Figure S1: Maps showing sampling sites. (*a*) Samples were collected at four sites on the mainland (Liverpool, Penryn, Le Conquet and Quiberon) and six islands in the Irish Sea (Arran and the Isle of Man), the English Channel (Scilly Isles, Alderney and Guernsey) and off the coast of France (Ouessant and Belle-Ile) in 2021. Six islands (coloured points) were additionally sampled in 2022 to study how population structure affects disease prevalence. Closeups (*b*) to (*f*), sorted from north to south show five sites on the Isle of Arran (*b*), five sites on the Isle of Man (*c*), Alderney and five sites on Guernsey (*d*), Ouessant (*e*) and five sites on Belle-Ile (*f*) that were sampled in 2022. Note that the scale in (*a*) is 200 km and 20 km in (*b*) to (*f*).

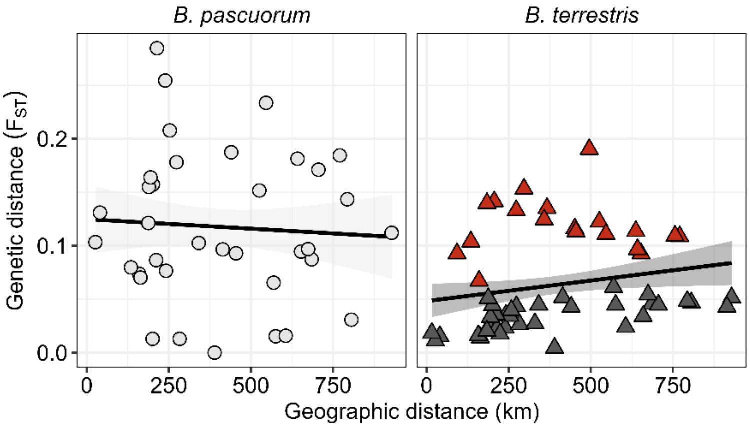

Figure S2: Genetic distance (F_{ST}) by geographic distance between the populations of *Bombus pascuorum* (left, light grey and circles) and *B. terrestris* (right, dark grey and triangles). For *B. terrestris*, pairwise distances that include the Isle of Man or the Scilly Isles are highlighted in red. The lines indicate a linear regression with 95% confidence intervals. A Mantel test showed no correlation between genetic and geographic distance, however, when excluding the Isle of Man and the Scilly Isles, there was a significant correlation between geographic and genetic distance in *B. terrestris* (r = 0.6203, p = 0.001).

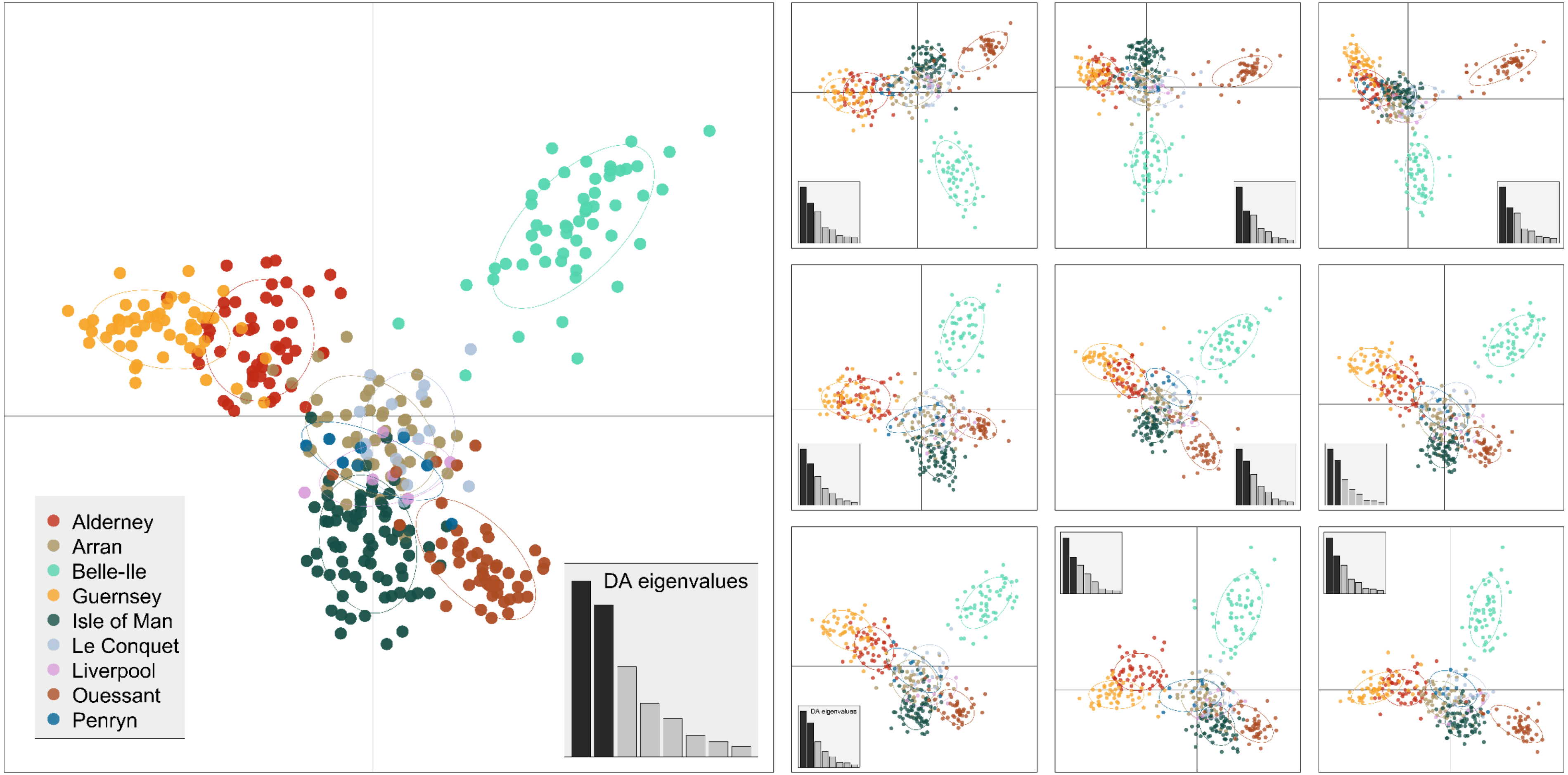

Figure S3: Scatterplots from ten random subsampled date sets showing discriminant analysis of principal components (DAPC) of the first two principal components discriminating *Bombus pascuorum* populations. Ellipses showing 67% confidence intervals are centred on each of the six islands (Arran, Alderney, Belle-Ile, Guernsey, Isle of Man and Ouessant; from 2021 and 2022) or three mainland (Le Conquet, Liverpool and Penryn; from 2021) sites. Individuals are shown as points. The eigenvalues of the analysis are displayed in the inset. Each plot shows one of the subsets with 50 randomly selected individuals from each population.

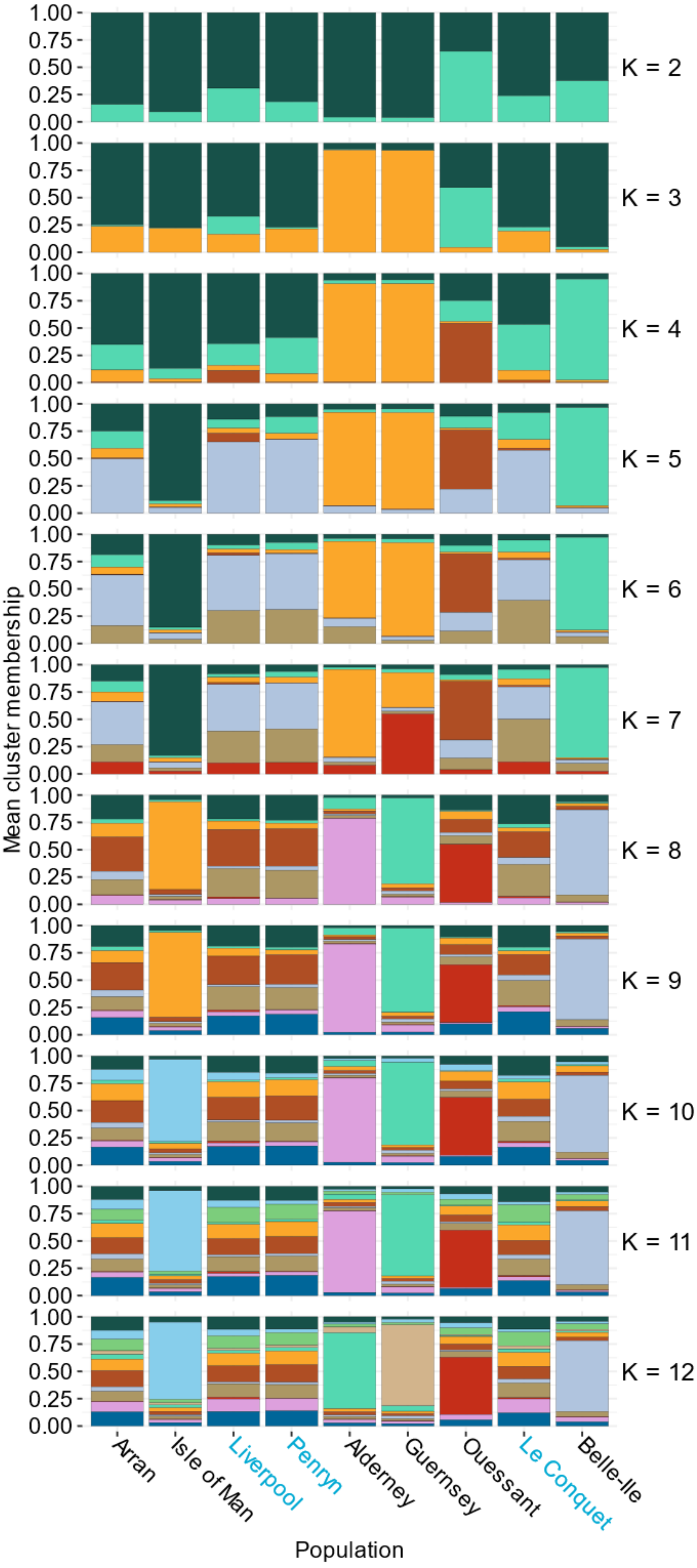

Figure S4: Proportional membership of *Bombus pascuorum* island (black labels) and mainland (blue labels) samples from 2021 and 2022 from K = 2 to K = 12 genetic clusters (as estimated in STRUCTURE). Average membership by population across 10 subsets with 15 individual bees (Liverpool n = 6 and Penryn n = 8) is shown. Populations are arranged from north to south and shown as bars with colours corresponding to the estimated relative membership in the different clusters. K = 5 is shown in Figure 1.

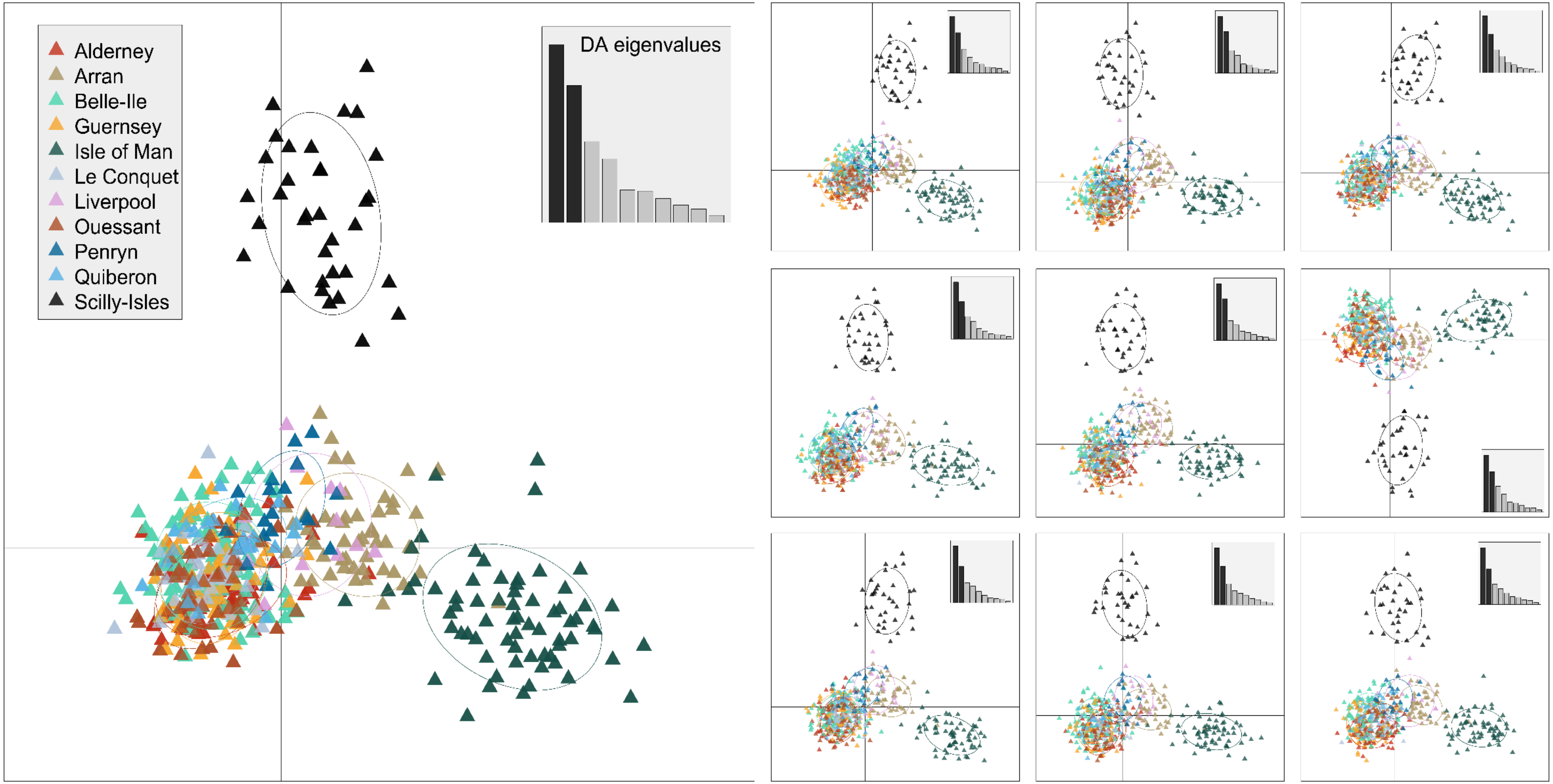

Figure S5: Scatterplots from ten random subsampled data sets showing the discriminant analysis of principal components (DAPC) of the first two principal components discriminating *Bombus terrestris* populations. Ellipses showing 67% confidence intervals are centred on each of the seven islands (Arran, Alderney, Belle-Ile, Guernsey, Isle of Man, Ouessant and the Scilly-Isles; from 2021 and 2022) or the four mainland (Le Conquet, Liverpool, Penryn and Quiberon; from 2021) sites. Individuals are shown as triangles. The eigenvalues of the analysis are displayed in the inset. Each plot shows one of the 10 subsets with 50 randomly selected individuals from each population.

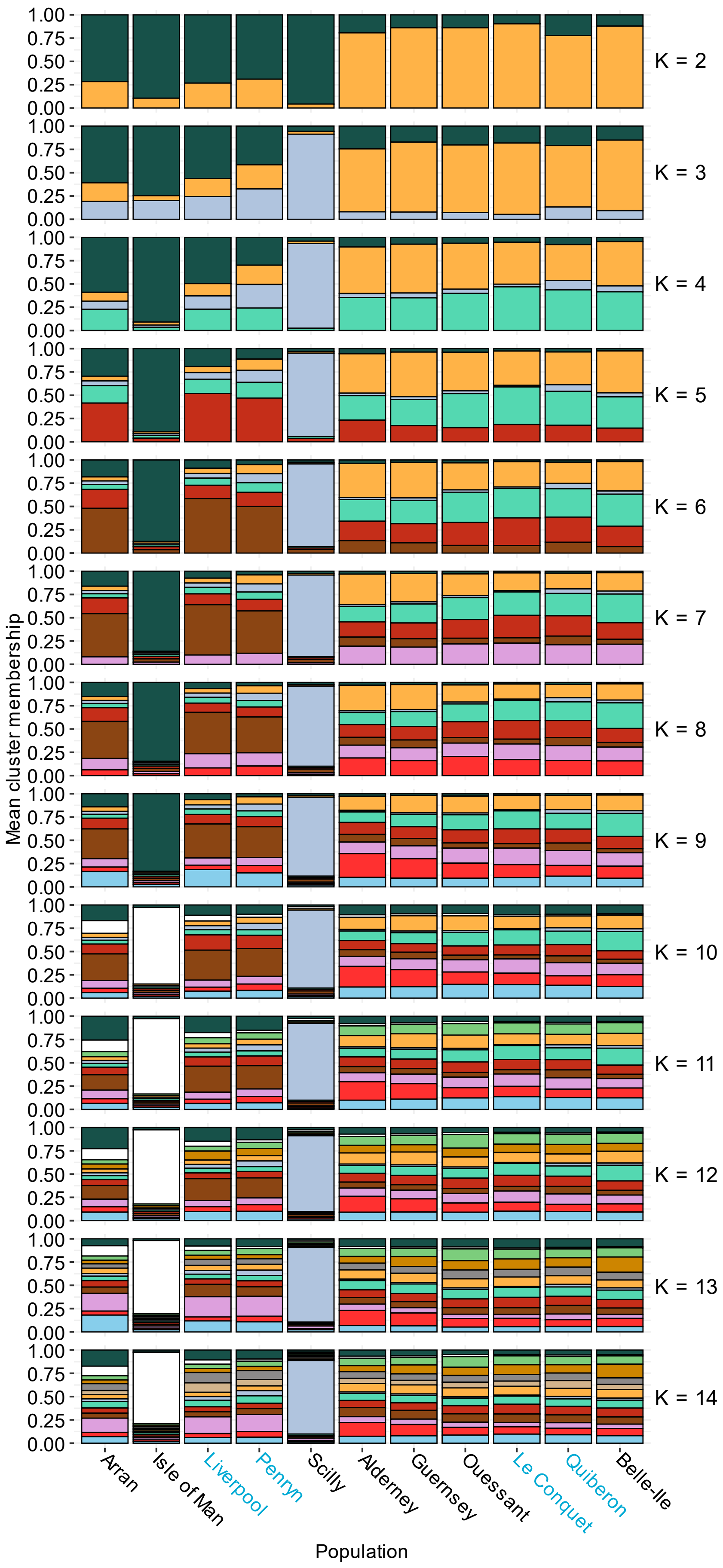

Figure S6: Proportional membership of *Bombus terrestris* island (black labels) and mainland (blue labels) samples from 2021 and 2022 from K = 2 to K = 14 genetic clusters (as estimated in STRUCTURE). Average membership by population across 10 subsets with 15 individual bees (except Liverpool n = 12) is shown. Populations are arranged from north to south and shown as bars with colours corresponding to the estimated relative membership in the different clusters. K = 4 is shown in Figure 1.

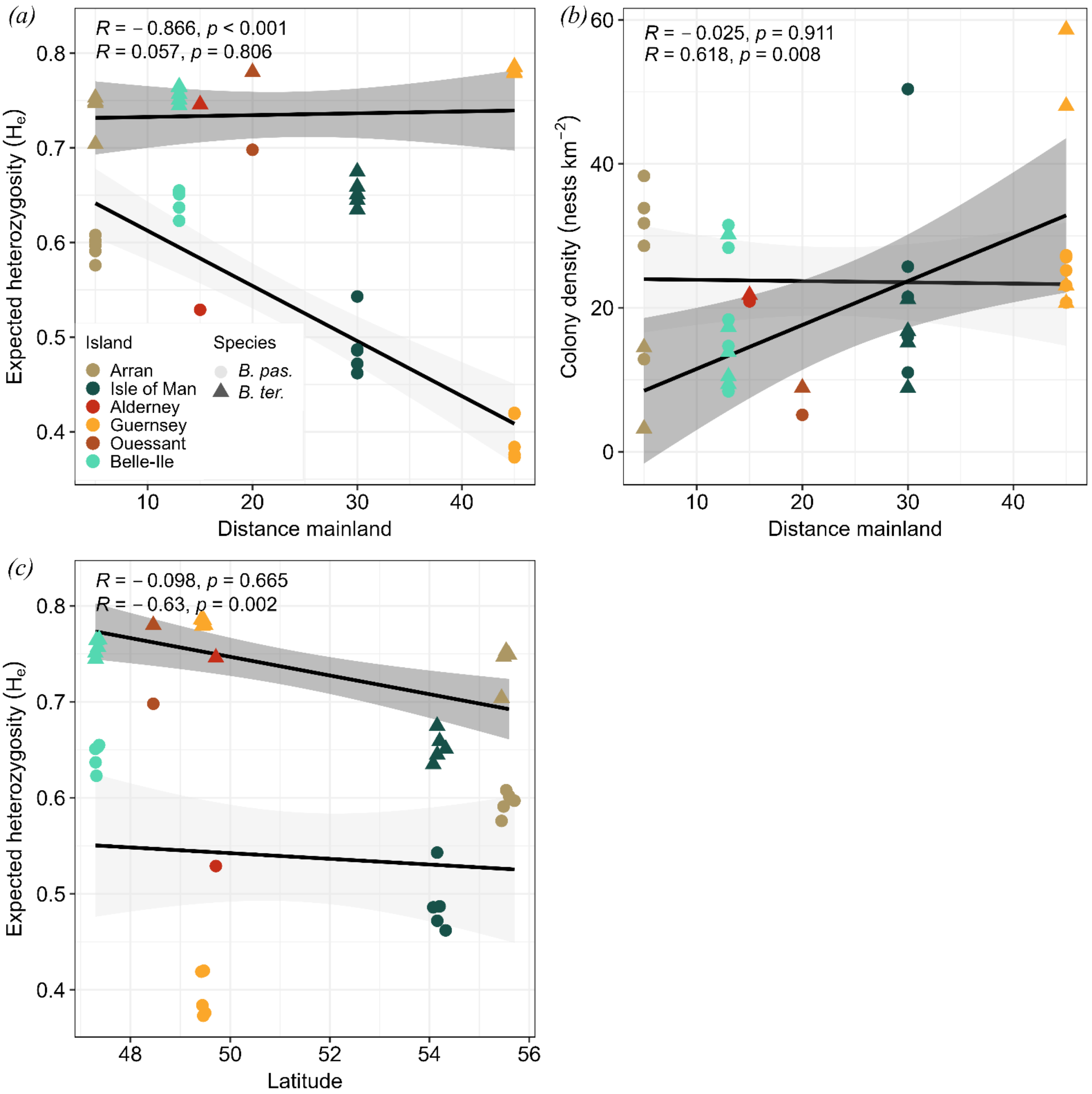

Figure S7: Scatterplot showing the correlation between *(a)* island distance from the mainland and expected heterozygosity (H_e), *(b)* island distance from the mainland and colony density or *(c)* latitude and expected heterozygosity (H_e) in *Bombus pascuorum* (circles) and *B. terrestris* (triangles). Shapes are coloured by the island and represent different sites on the island (or different collection time points for Alderney). The lines indicate a linear regression with 95% confidence intervals for *B. pascuorum* (light grey) and *B. terrestris* (dark grey). Pearson's correlation tests are shown for *B. pascuorum* (top) and *B. terrestris* (bottom).

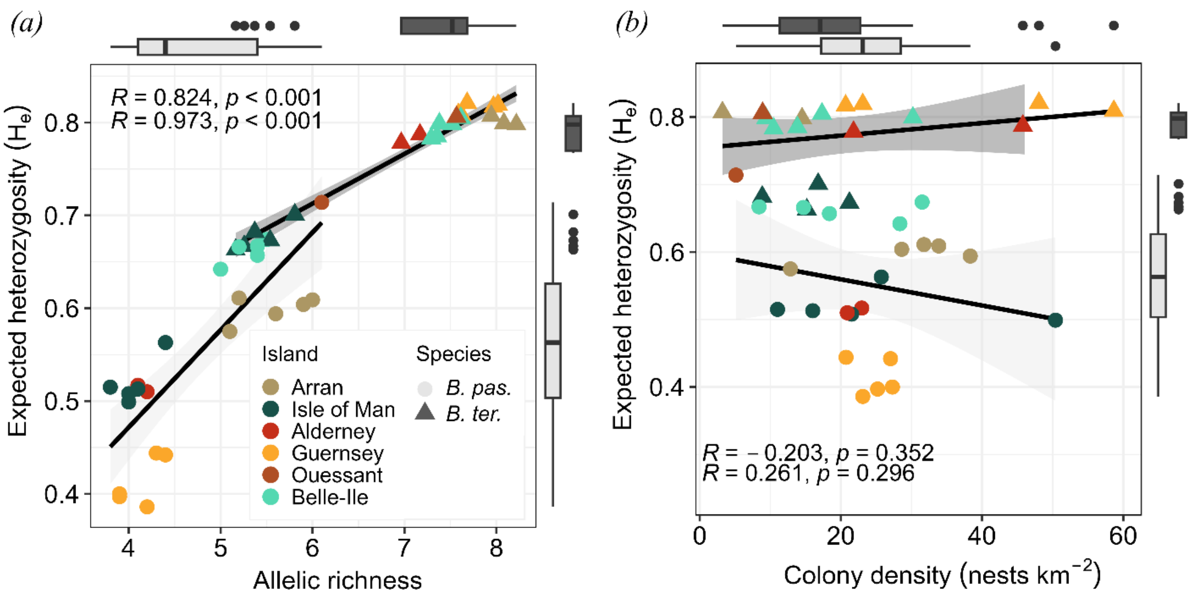

Figure S8: Scatterplot showing a strong correlation between *(a)* allelic richness (AR) and expected heterozygosity (H_e) and between *(b)* colony density and H_e in *Bombus pascuorum* (circles) and *B. terrestris* (triangles). Shapes are coloured by the island and represent different sites on the island (or different collection time points for Alderney). The lines indicate a linear regression with 95% confidence intervals and the boxplots on the side show AR, colony density and H_e by species. The Pearson's correlation tests show a significant correlation between AR and $H_{e,}$ but not between nest density and H_e for both species.

References:

Dobelmann, J., Manley, R., & Wilfert, L. (2024). Caught in the act: the invasion of a viral vector changes viral prevalence and titre in native honeybees and bumblebees. *Biology Letters, 20*(5), 20230600. doi:10.1098/rsbl.2023.0600

Estoup, A., Solignac, M., Cornuet, J. M., Goudet, J., & Scholl, A. (1996). Genetic differentiation of continental and island populations of *Bombus terrestris* (Hymenoptera: Apidae) in Europe. *Molecular Ecology, 5*(1), 19-31. doi:10.1111/j.1365-294X.1996.tb00288.x

Estoup, A., Tailliez, C., Cornuet, J.-M., & Solignac, M. (1995). Size homoplasy and mutational processes of interrupted microsatellites in two bee species, *Apis mellifera* and *Bombus terrestris* (Apidae). *Molecular Biology and Evolution, 12*(6), 1074-1084. doi:10.1093/oxfordjournals.molbev.a040282

Fries, I., Chauzat, M.-P., Chen, Y.-P., Doublet, V., Genersch, E., Gisder, S., . . . Williams, G. R. (2013). Standard methods for Nosema research. *Journal of Apicultural Research, 52*(1), 1-28. doi:10.3896/IBRA.1.52.1.14

Manley, R., Temperton, B., Boots, M., & Wilfert, L. (2020). Contrasting impacts of a novel specialist vector on multihost viral pathogen epidemiology in wild and managed bees. *Molecular Ecology, 29*(2), 380-393. doi:10.1111/mec.15333

Meeus, I., de Miranda, J. R., de Graaf, D. C., Wackers, F., & Smagghe, G. (2014). Effect of oral infection with Kashmir bee virus and Israeli acute paralysis virus on bumblebee (*Bombus terrestris*) reproductive success. *Journal of Invertebrate Pathology, 121,* 64-69. doi:10.1016/j.jip.2014.06.011

Puechmaille, S. J. (2016). The program structure does not reliably recover the correct population structure when sampling is uneven: subsampling and new estimators alleviate the problem. *Molecular Ecology Resources, 16*(3), 608-627. doi:10.1111/1755-0998.12512

Reber Funk, C., Schmid-Hempel, R., & Schmid-Hempel, P. (2006). Microsatellite loci for *Bombus* spp. *Molecular ecology notes, 6*(1), 83-86. doi:10.1111/j.1471-8286.2005.01147.x

Schmid-Hempel, R., & Tognazzo, M. (2010). Molecular divergence defines two distinct lineages of *Crithidia bombi* (Trypanosomatidae), parasites of bumblebees. *Journal of Eukaryotic Microbiology, 57*(4), 337-345. doi:10.1111/j.1550-7408.2010.00480.x
